# The Roles of Dispersal, Fecundity, and Predation in the Population Persistence of an Oak (*Quercus engelmannii*) under Global Change

**DOI:** 10.1371/journal.pone.0036391

**Published:** 2012-05-18

**Authors:** Erin Conlisk, Dawn Lawson, Alexandra D. Syphard, Janet Franklin, Lorraine Flint, Alan Flint, Helen M. Regan

**Affiliations:** 1 Department of Biology, Center for Conservation Biology, University of California Riverside, Riverside, California, United States of America; 2 Environmental Sciences and Applied Systems Branch, Space and Naval Warfare Systems Center Pacific, San Diego, California, United States of America; 3 Conservation Biology Institute, La Mesa, California, United States of America; 4 School of Geographical Sciences and Urban Planning, Arizona State University, Tempe, Arizona, United States of America; 5 USGS California Water Science Center, Sacramento, California, United States of America; Ohio State University, United States of America

## Abstract

A species’ response to climate change depends on the interaction of biotic and abiotic factors that define future habitat suitability and species’ ability to migrate or adapt. The interactive effects of processes such as fire, dispersal, and predation have not been thoroughly addressed in the climate change literature. Our objective was to examine how life history traits, short-term global change perturbations, and long-term climate change interact to affect the likely persistence of an oak species - *Quercus engelmannii* (Engelmann oak). Specifically, we combined dynamic species distribution models, which predict suitable habitat, with stochastic, stage-based metapopulation models, which project population trajectories, to evaluate the effects of three global change factors – climate change, land use change, and altered fire frequency – emphasizing the roles of dispersal and seed predation. Our model predicted dramatic reduction in *Q. engelmannii* abundance, especially under drier climates and increased fire frequency. When masting lowers seed predation rates, decreased masting frequency leads to large abundance decreases. Current rates of dispersal are not likely to prevent these effects, although increased dispersal could mitigate population declines. The results suggest that habitat suitability predictions by themselves may under-estimate the impact of climate change for other species and locations.

## Introduction

Species distribution models (SDMs), which predict suitable habitat as a function of environmental conditions [1], have been widely used in conservation to estimate suitable habitat under climate change. Observations and predictions show climatically suitable habitat shifting in response to a warming climate, typically moving to higher elevations and latitudes [2], [3], [4]. Species’ ability to avoid extinction depends in part on their ability to disperse from less to more suitable habitat [5]. Studies relying on SDMs and related methods have estimated how far certain species may need to migrate, but there is considerable uncertainty about whether plants can do so [6]. Comparisons of C^14^-dated pollen sequences against models of long-distance dispersal suggest that species may have been restricted historically by climate and not dispersal [7]. However, accelerated rates of current climate change may make migration impossible [8]. Combined with the uncertain impact of habitat fragmentation on dispersal and varying dispersal abilities, there is little consensus concerning the ability of species to keep pace with climate change.

Previous studies of the ability of species to keep up with climate change have typically focused on the distances between current and future suitable habitat [9]. Dispersal was presumed to occur if current and future suitable habitat predictions overlapped or the distances between them were small [5], [10]. Alternatively, the projected rate of change in time and space of topoclimatic environments can be estimated, thus describing the velocity that species within an ecosystem would have to travel to keep pace with climate change [11]. It was found that flat areas (low topographic relief) would require the largest velocities. Nevertheless, these various approaches did not directly address how species would accomplish the migration. Other studies have modeled life history traits and demographic dynamics explicitly through population spread models of wind-dispersed trees [12], [13]. Although these studies modeled realistic dispersal functions that include migration of individual propagules, they did not incorporate future habitat suitability. Our approach is to combine elements of both approaches to simultaneously model climate-induced change in metapopulations (spatially separated populations interacting through migration) and demographic processes of seed dispersal between these populations.

For plant species, fecundity and mode of dispersal underpin migration. Fecundity determines the number of available propagules while dispersal mode influences the distance and frequency of dispersal, namely the dispersal kernel. Both are expected to change with climate change, with potentially more complicated changes occurring in species, like oaks, that mast (i.e. exhibit regionally synchronized low seed production in most years and high seed production in occasional years). Acorn production has been shown to be temperature sensitive in *Quercus crispula* [14] and moisture sensitive in *Q. ilex* [15]. Maturation timing of acorns and adults may also change with climate change [16], with direct effects on survival and indirect effects on dispersal agents and seed predator avoidance [17]. Depending on how climate change impacts dispersal agents, masting events may cause decreased dispersal distances for some species ([18] for *Quercus*, [19] for tropical trees), but increased dispersal distances for others ([20] for pines). Decreased dispersal distance need not result in higher near-parent germination and establishment since higher overall seed survival and seedling density can preferentially occur at greater dispersal distances in *Quercus* masting years [18]. Uncertainty about the future relative proportions of different seed predators makes it difficult to predict the impact of climate change.

In addition to impacts on future habitat suitability, dispersal capacity, and seed predators, climate change may also affect species’ persistence indirectly by altering ecosystem processes such as fire, which is especially important in Mediterranean ecosystems [21]. The mechanism for these potential effects would likely be climatic influences on fuel moisture, fuel load, and ignition probability [22]. Increases in fire frequency are also strongly associated with land use change and human population growth, particularly in rural areas [21], [23], [24]. Thus, we expect interactions among climate change, land use change, and altered fire regimes. For example, if habitat fragmentation decouples fires (effectively reducing fire size and thus the total area burned across habitat patches), then there may be an optimal degree of fragmentation for an obligate seeding population (plants requiring fire to germinate, but sensitive to frequent fires) [25]. Other studies found that altered fire frequency coupled with climate change could decrease obligate seeder populations [26], [27]. However, no previous studies have included detailed masting, dispersal, and seed predation dynamics in demographic predictions under global change. Further, few studies address the impact of altered fire frequency coupled with climate and land use change on fire resprouters (species with persistent individuals that resprout after a fire) despite numerous studies showing the minimal impact of high fire frequency on resprouters [24], [28]. Because altered fire regimes have the potential to dramatically alter species composition and ecosystem processes, understanding the relative role of fire in combination with other global change processes is essential to conservation management.

Here we study a resprouting oak species of conservation interest, *Quercus engelmannii* (Engelmann oak). It is a small (10-meter tall) white oak, endemic to the south coast montane section of the California floristic province. It is classified on the IUCN Red List as vulnerable, primarily due to habitat loss [29]. This emblematic oak is ecologically important, with acorns providing food resources for hundreds of species of insects and at least 100 species of birds and mammals. There is concern that *Q. engelmannii* are not regenerating adequately, possibly due to invasive plants, fire, and grazing of cattle [30], [31]. Insufficient oak recruitment is not unique to *Q. engelmannii*, but has been noted in many Mediterranean *Quercus* species [32]. Thus, forecasting potential impacts of climate change for slowly-regenerating *Quercus* species requires careful consideration of disturbance and life history traits, in addition to habitat suitability. Previous work on future oak habitat suitability has been criticized for neglecting life history [4], [33].

Land use change and altered disturbance regimes are considered to be primary threats to biodiversity in Mediterranean ecosystems [34], [35]. Because these threats, along with climate change, are expected to continue, we seek to understand the role of dispersal, fecundity (especially masting), and seed predation in population dynamics under the influence of climate change, land use change and altered fire frequency for a long-lived obligate resprouter, *Q. engelmannii*. By simultaneously modeling habitat suitability and demography, our approach allows us to answer critical questions that were not addressed by previous models, namely, how do life history traits, short-term global change perturbations (such as fire frequency and land use change), and long-term climate change interact to affect the likely persistence of oaks? Specifically, we ask: Which threats are the most serious for *Q. engelmannii* conservation? Do the threats reinforce each other? How important is dispersal to *Q. engelmannii* populations? Does (i) rare long distance dispersal or (ii) number of propagules have a greater influence on population persistence? How do masting and seed predation affect population persistence?

## Methods

The overall modeling strategy is to use SDM predictions of habitat suitability over current and future climate to define the locations and carrying capacities of metapopulation patches, and to use a demographic model to determine the population dynamics within these patches. This approach to studying climate change, by linking dynamic bioclimatic envelopes with population models, is gaining traction within the conservation community [26], [27], [36].

### 

#### Current and future habitat distribution maps

**Figure 1:**
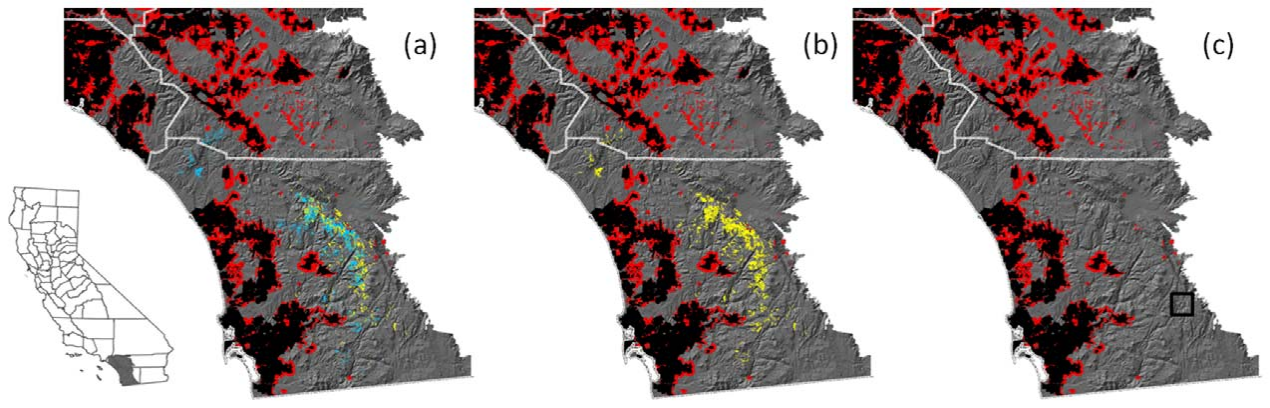
Map of the study area: most of San Diego and Orange Counties, western Riverside County, and the southern corners of Los Angeles and San Bernardino Counties (see inset). Map (a) shows habitat currently occupied by *Q. engelmannii* in cyan, and other habitat estimated to be currently suitable in yellow. Map (b) shows predicted 2100 suitable habitat in yellow for the PCM future climate scenario. Map (c) shows the same for the GFDL scenario (the box in the lower right surrounds the two small patches of remaining suitable habitat). In all three maps, the black represents current urban areas, and the red represents the extent of urban expansion by 2050.

Current distribution data (Fig. 1a) for *Q. engelmannii* was obtained from two sources: mapped habitat from the California Vegetation Mapping Program (CALVEG) [37] vegetation map (100-m resolution) and 139 presence locations from herbarium records and the 1930s Wieslander surveys [38]. Only three small patches (<5 ha) of *Q. engelmannii* remain in Los Angeles County (not shown in Fig. 1a).

We used Maxent presence-only species distribution modeling [39] to create suitability maps. Environmental predictors included climate (January minimum temperature, July maximum temperature, and annual precipitation), soil, and terrain variables important to predicting Southern California plant species distributions (see [40] and Appendix S1.2). Current climate grids were derived from 1971–2000 averaged Parameter-Elevation Regressions on Independent Slopes Model data (PRISM) [41] and spatially downscaled to a Digital Elevation Model [42]. The resolution of our climate, soil and terrain variables was 100 m. The resulting Maxent habitat suitability predictions provided a suitability index for *Q. engelmannii* in each of the one-hectare pixels. The training accuracy of the resulting current-climate suitability maps was AUC (area under the curve)  = 0.88. This suggests a reasonably accurate discrimination of occupied habitat [1]. The predicted distribution of currently suitable habitat was compared to maps of designated *Q. engelmannii* habitat from CALVEG [37].

We used one land-use change scenario, one emission scenario (A2, which assumes business as usual CO_2_ emissions in a socio-economically heterogeneous world), and two general circulation model projections (the Department of Energy and National Center for Atmospheric Research’s Parallel Climate Model, or PCM, and the National Oceanic and Atmospheric Association’s Geophysical Fluid Dynamic Laboratory’s CM.2 model, or GFDL). PCM was generally less sensitive to climate forcings and predicted a slightly hotter and wetter climate, and the more sensitive GFDL predicted a substantially hotter and drier climate for California. These climate models represent two very different precipitation scenarios and are the preferred models for California because their predictions of historic climate match observations [43]. Climate variables for 2070–2099 were averaged to represent predicted climate ca. 2099 for each climate scenario.

We created future habitat suitability maps for each climate projection by substituting future climate data in the suitability function obtained using the current climate. To create a time series of dynamic habitat maps across 100 years, we linearly interpolated habitat suitability in each cell over the model time horizon. This resulted in two sets (for PCM and GFDL projections) of 50 maps of suitable habitat for two year time-steps for 2000–2099.

To create dynamic projections of urban growth for 2000–2050, we developed spatially explicit, binary projections of urban development using the SLEUTH model [44]. When predicted urban development overlapped suitable habitat, these areas were considered unsuitable (see Fig. 1). Predictions of land use change beyond the next 50 years would be unduly speculative [45]. While urbanization has accelerated since 1920 [45], the rate of future urban growth is predicted to slow in the next 50 years due to lack of available land [44].

A patch-structured metapopulation map was constructed by selecting a threshold suitability value (*p* = 0.75) above which predicted suitability was retained and below which suitability was set to zero. The threshold was chosen such that the area predicted to be suitable was roughly equal to the area observed to be occupied [46]; thus, 15,000 ha of the CALVEG designated habitat was deemed suitable plus an additional 12,000 ha to account for potentially suitable areas that may be currently unoccupied. Habitat suitability maps were imported into the spatially explicit population modeling platform RAMAS GIS® 5.0 [47]. Suitable patches were defined as clusters of 15 or more adjacent suitable one-hectare pixels. Smaller patches would have violated RAMAS limits on the total number of metapopulation patches; and sensitivity analyses exploring the deletion and addition of small patches showed that adding patches less than 15 hectares did not alter results. The carrying capacity of a patch was calculated as the sum of habitat suitabilities over all cells within the patch multiplied by a maximum density of 150 adult trees per hectare-equivalent [48]. The term “hectare-equivalent” reflects the fact that two patches with the same carrying capacity can have different areas due to differing habitat suitabilities within a patch.

#### Stage transitions in the demographic model

Five life stages were assumed: acorn, small seedling, large seedling, sapling, and adult tree. These stages were chosen to match available data from field experiments describing the average and standard deviation of transition rates of seedlings and saplings in the presence and absence of a fire over a two-year time step [49]. Data from the published literature were used to estimate values for all other parameters (see Appendix S1 for details). Time-steps were defined to be two years to match the observation interval of the field data (see Appendix S1.3). Vital rates depend on fire, time since last fire, masting, carrying capacity constraints, random disturbances and natural variability. In a baseline deterministic case when dispersal is absent and fire, masting, and capacity constraints do not apply, the life-stage populations in each patch would be governed by:
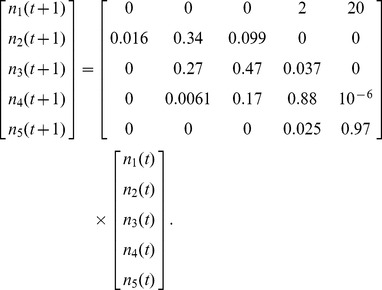
(1)Here the *n_i_*(*t*) refer to the populations, by stage, ordered by increasing size, at time-step *t*. The numerical matrix shows the specified mean values of the vital rates. The first row of the matrix represents fecundities over a two-year time-step. The remaining entries represent (two-year) probabilities of survival (diagonals) and stage transitions (off-diagonals). For example, element (2, 3) in (1) describes the probability that a large seedling would be damaged and thus become a small seedling. Since 0.97^100^ ≈ 0.05, the adult survival probability of 0.97 corresponds to approximately a 5% chance of individuals living longer than 100 time-steps (200 years) [50].

The baseline case of eqn 1 is an expository starting point. The full model includes environmental and demographic stochasticity, disturbance and masting events, density dependence, response to fire, and patch dynamics. Different patches were interconnected by dispersal of acorns. New patches formed and old patches dissolved according to changes in suitable habitat. Realized vital rates in each time step and patch were randomly perturbed about their means to account for environmental stochasticity. During patch-specific episodes of fire, masting, and/or carrying capacity exceedance, the mean vital rates changed. Thus, the metapopulation consisted of many stage-based population vectors *n_i_*(*t*), one for each patch and time-step, changing according to multiple biological mechanisms, random disturbances, and stochasticity.

A first departure from the baseline case was the imposition of environmental and demographic stochasticity on the vital rates. For environmental stochasticity, each vital rate in each patch at each time-step was replaced by an independent draw from a lognormal distribution using the mean shown in eqn 1 and corresponding standard deviation [49] (see Appendix S1.3):
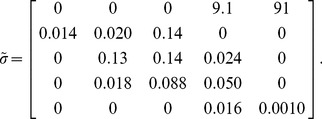
(2)The lognormal distribution allows for positive transition rates with fat tails towards higher survival probabilities. All survival probabilities were truncated at one. The vital rates thus drawn were used as patch-specific means to impose demographic stochasticity via independent draws from a Poisson distribution (for fecundities) or a multinomial distribution (for transition rates).

### Fecundity, Masting, and Acorn Predation

Visual in-tree *Q. engelmannii* acorn counts from the California Acorn Survey Project (Koenig, personal communications) were translated into canopy level fecundity estimates using a regression for *Q. douglasii* in [51] (see Appendix S1.5). The effective number of viable acorns was the product of acorn production and survival rates from multiple seed predators. Seed predation was assumed to occur prior to dispersal and post-dispersal predation was implicit in germination rates. For predation of *Q. ilex* acorns by insects [52], and for predation of *Q. rubra, Q. alba*, and *Q. palustris* acorns by small vertebrates [18], the proportion of acorns killed varied according to the number of acorns produced, such that masting years had lower proportional acorn predation. We used data from these studies to determine predation rates, assuming that some of the acorns reported as “predated” were actually buried, thus facilitating germination. We converted fecundity estimates from one year time-steps to two year time-steps. Although these studies were performed on other *Quercus* species, the use of collateral data from similar species is a well-established practice when data for a target species are limited [53].

We developed six scenarios describing the number of viable acorns by combining three masting assumptions with two predation assumptions (see Table 1 and Appendix S1.5 for details). The three masting assumptions were no masting, masting with probability 0.121 in each time-step, and masting with probability 0.527 in each time-step. In the first predation assumption, predation was lower in masting time-steps (approximately 96% predation of acorns) than non-masting time-steps (approximately 99% predation). In the second predation assumption, predation was approximately 96% in both masting and non-masting time-steps. These masting frequencies were based on 16 years of *Q. engelmannii* acorn productivities (Koenig, personal communication), and on masting frequencies reported in [54]. Although we do not consider episodic bursts in germinability, our masting scenarios lead to episodic recruitment, which may occur in *Quercus* species [50].

**Table 1:** Six masting-predation scenarios.

Non-masting predation rate	Mastingpredation rate	Masting probability per time-step	Number of viable acorns per non-masting time-step	Number of viable acorns per masting time-step	Coefficient of variation in acorns per time-step
0.992	0.965	0.527	20	162	0.95
0.991	0.963	0.121	30	271	0.93
0.991	na	0	36	Na	1.19
0.965	0.965	0.527	82	187	1.00
0.963	0.963	0.121	121	316	0.93
0.967	na	0	127	Na	1.19

The top three rows list the scenarios in which seed predation is lower in masting years. These first three rows are ordered by decreasing masting frequency. The bottom three rows list scenarios for which seed predation does not vary between masting and non-masting years. The last three rows are ordered by decreasing masting frequency. The coefficient of variation is the same in masting and non-masting years.

### Fire

For each patch and two-year time-step, the probability of fire was assumed to depend on time since last fire according to a discrete time Weibull hazard function:
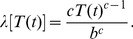
(3)Here *λ*[*T*(*t*)] denotes the probability of a fire at time-step *t* given that the last fire occurred *T*(*t*) time-steps earlier; and *b* and *c* are scale and shape parameters [55]. We set *c* = 1.42, suggesting a relatively low influence of time since last fire as is common in chaparral [55]. In simulations, we chose *b* to represent average fire return intervals of 20, 30, 40, 50, 60, 70, 80, and 120 years. There was also a no-fire scenario. Future fire frequency is highly uncertain because land use change will likely alter fire ignitions and climate change will likely alter fuel build up, fuel moisture, temperature, and precipitation. Thus, we explore a range of fire frequencies, noting that historic fire frequencies in San Diego County typically range from 10–90 years for a variety of vegetation types [56]. At the start of a simulation, each patch was given an initial value *T*(0) drawn from the Weibull distribution. Fires were assumed to be spatially independent and to burn entire patches. The largest patch in our model was approximately 4,600 ha, much smaller than the six largest (>100,000 ha) southern California fires that have occurred since 2001.

In a time-step when a fire occurs, the mean vital rates matrix changes to a fire matrix:
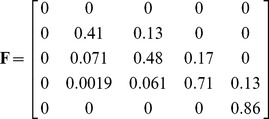
(4)Eqn 4 reflects research showing that adults resprout from canopy crowns, saplings resprout vigorously from basal root crowns, small seedlings rarely resprout, and large seedlings resprout more than small seedlings but less than saplings [30]. We assumed germination and fecundity to be zero in a fire time-step, with fecundity recovering completely to eqn 1 rates by the next time-step [57]. To reflect the lingering negative effects of fire on *Quercus* germination and seedling recruitment [48], for seven years following a fire we halved the germination rate relative to its eqn 1 value, akin to observed germination decreases due to canopy opening from coppicing [58]. Similarly, post-fire large seedling and sapling survival transition rates were increased by 10% to reflect the positive impact of canopy opening on these stages (see Appendix S1.7).

### Dispersal

Dispersal was assumed to depend on the distance between the centers of two patches. Although mice and squirrels disperse acorns over small within-patch distances, birds, in particular jays, were assumed to do the bulk of between-patch dispersal [59], [60]. Acorn woodpeckers live in territorial groups with arboreal hoards containing many acorns; thus they typically do not contribute to long-range dispersal [61]. The probability that a seed migrates from a patch *i* to another patch *j* was assumed to be:
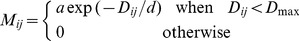
(5)where *D_ij_* is the distance between the patches; *D*_max_ is the maximum possible dispersal distance; and *a* and *d* are fixed parameters determining the maximum rate of dispersal and the rate of dispersal decline with distance. The coefficient *a* might be interpreted as the distance-independent parameter of acorn flow. When the maximum dispersal distance is sufficiently large, *d* can be interpreted (by reference to the mean of an exponential distribution) as the average dispersal distance. For any row *i* of the symmetric matrix of *M_ij_* values, the row sum equals the expected total fraction of acorns leaving patch *i* and, thus, may not exceed one. We use an exponential dispersal kernel because it fit empirical data of jay dispersal in oak woodlands with a high degree of statistical significance [59].

Because of the uncertainty in current and future dispersal distances, we explored a variety of dispersal parameters (*a*, *d*, *D*_max_) guided by the few available empirical studies. The maximum dispersal distance observed in the literature was 4-km for scrub jays (*Cyanocitta cristata*) in pin oak (*Q. palustris*) forests [62]. Studies observed average dispersal distances of 1.1 km for scrub jays in pin oak forests [63] and 0.263 km for European jays (*Garrulous glandarius*) in Holm oak (*Q. ilex*) forests [59]. Although genetic analyses of relatedness suggest limited long-range oak dispersal [64], results from preliminary simulations were pessimistic about the persistence of *Q. engelmanii* in the absence of long-range dispersal. Thus, we emphasized high dispersal distances to determine what might, in principle, mitigate population decline. Acorn “flow”, or *a*, was varied from 0.0001 to 10. Average dispersal distance, *d*, was varied from 0.3 to 10 km and maximum dispersal distance, *D_max_*, was varied from 4 to 40 km.

### Carrying Capacity Constraints

Each patch had its own carrying capacity, determined by habitat suitability and habitat area. When, as a result of fecundity or dispersal, a patch’s abundance exceeded its capacity, survival rates (diagonal elements in the matrix of eqn 1) and stage-growth rates (subdiagonal elements in the matrix of eqn 1) were temporarily decreased to force a gradual decline in abundance, back to capacity (see Appendix S1.9). Individuals at the five life stages (acorns to adults) were assumed to contribute to patch abundance with stage-weights (0, 0.0025, 0.025, 0.25, 1). The carrying capacity constraint required that abundance not exceed 150 adult-equivalent plants/hectare-equivalent. At the start of a simulation, habitable patches were assigned initial population densities of 90 adult-equivalents/ha, with a distribution by stage equal to an average distribution over trial runs.

### Model Scenarios

We investigated a range of model scenarios, combining three possible climate projections (no change, PCM climate change projections, and GFDL climate change projections), two land use scenarios (no change or land use change), nine afire frequencies, 30 dispersal scenarios, and six coupled masting and seed predation scenarios. Each model was run 1000 times, with each run lasting 120 time steps. Stage and patch abundances were recorded at each time step. Expected minimum abundance (EMA), estimated as the average of the minimum abundances for the 1,000 model runs, was used to compare results across models. Expected minimum abundance effectively measures risk to population persistence because it is robust to parameter changes and captures the central tendency in the population’s lower extreme [65]. There was little variation (less than 1% of reported values) among repeated 1000-run simulations.

### Sensitivity Analyses

Beyond our analyses of different dispersal distances, masting scenarios, and fire return intervals, we performed sensitivity analyses in vital rates by individually increasing each element by 10% over the mean reported in eqn 1. For this analysis we considered two fire return intervals (fires every 20 years and no fires) and three dispersal scenarios (no dispersal, *d* = 1 and *D*_max_ = 4, and *d* = 10 and *D*_max_ = 20) for the PCM climate change scenario (Appendix S2.1). The vital rate with the greatest uncertainty was the germination parameter. Germination can vary from year to year due to a variety of environmental factors (discussed in Appendix S1.6). Thus, we did additional analyses increasing and decreasing that parameter by 50% and considering all fire return intervals (Appendix S2.2). Finally, we considered dispersal as a function of the carrying capacity of the receiving patch (Appendix S2.3).

## Results

**Figure 2:**
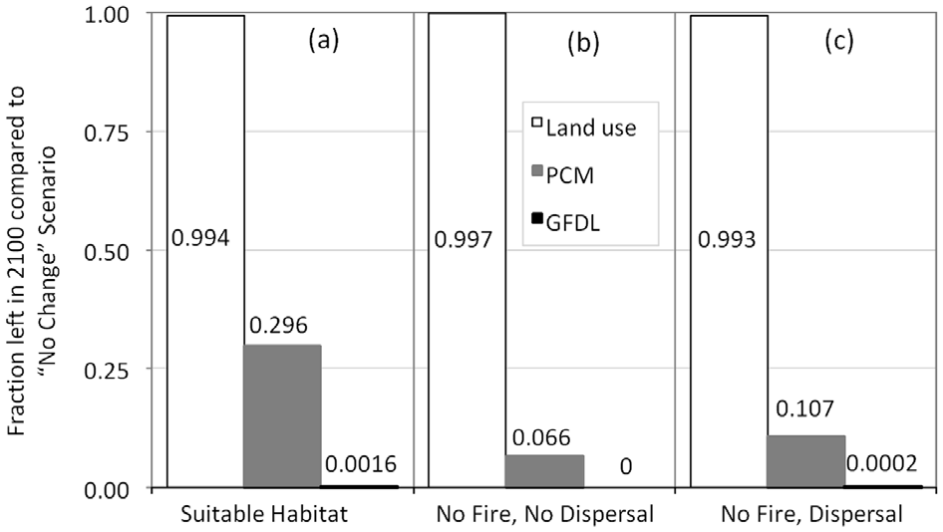
*Q. engelmannii* habitat ratios and population ratios under various assumptions. (a) Suitable habitat areas predicted for 2100 as ratios to suitable habitat areas estimated for 2000. The ratios are based only on habitat suitability maps. (b) The demographic component of the model is added, but with no fire and no dispersal. Each bar represents a ratio of a 2100 predicted population under a land use or climate change scenario to the 2100 predicted population under the no change scenario (where, in the no change scenario, suitable habitat is the same in 2000 and 2100). (c) Dispersal is added as described in text eqn 5, with the high values of average dispersal distance *d* = 10 km, and maximum dispersal distance *D*_max_ = 20 km, and with the flow parameter *a* = 0.01. All calculations for this figure assume the absence of fire.

Predicted suitable habitat for *Q. engelmannii* in 2100, under both the PCM and GFDL climate scenarios, was dramatically reduced in comparison to currently occupied habitat (Fig. 1). Climatically suitable habitat was predicted to shrink in extent and move southeastward to higher elevations in east San Diego County. Since these habitats are not likely to be diverted to urban development in the next 100 years, predicted land use change did not exert much influence on suitable habitat (Fig. 2). Nearly all suitable habitat remained after urban growth, whereas only 29.6% of suitable habitat remained under the PCM scenario, and almost no suitable habitat remained (0.16%) under the GFDL scenario (Fig. 2a).

When population dynamics, in addition to habitat changes, were considered in the absence of fire, the general pattern remained the same. Under a no-dispersal scenario, the expected minimum abundance (EMA) under land use change was 99.7% of the EMA for no habitat change, while the EMA under the PCM and GFDL scenarios was 6.6% and 0% (respectively) of the EMA for no habitat change (Fig. 2b). EMAs improved slightly when dispersal was added, with EMAs unchanged under land-use change relative to no habitat change, and 10.7% and 0.02% (respectively) for the PCM and GFDL scenarios relative to no habitat change (Fig. 2c). Dispersal allowed *Q. engelmannii* to follow the migrating habitat, thus slightly mitigating projected climate change effects.

**Figure 3:**
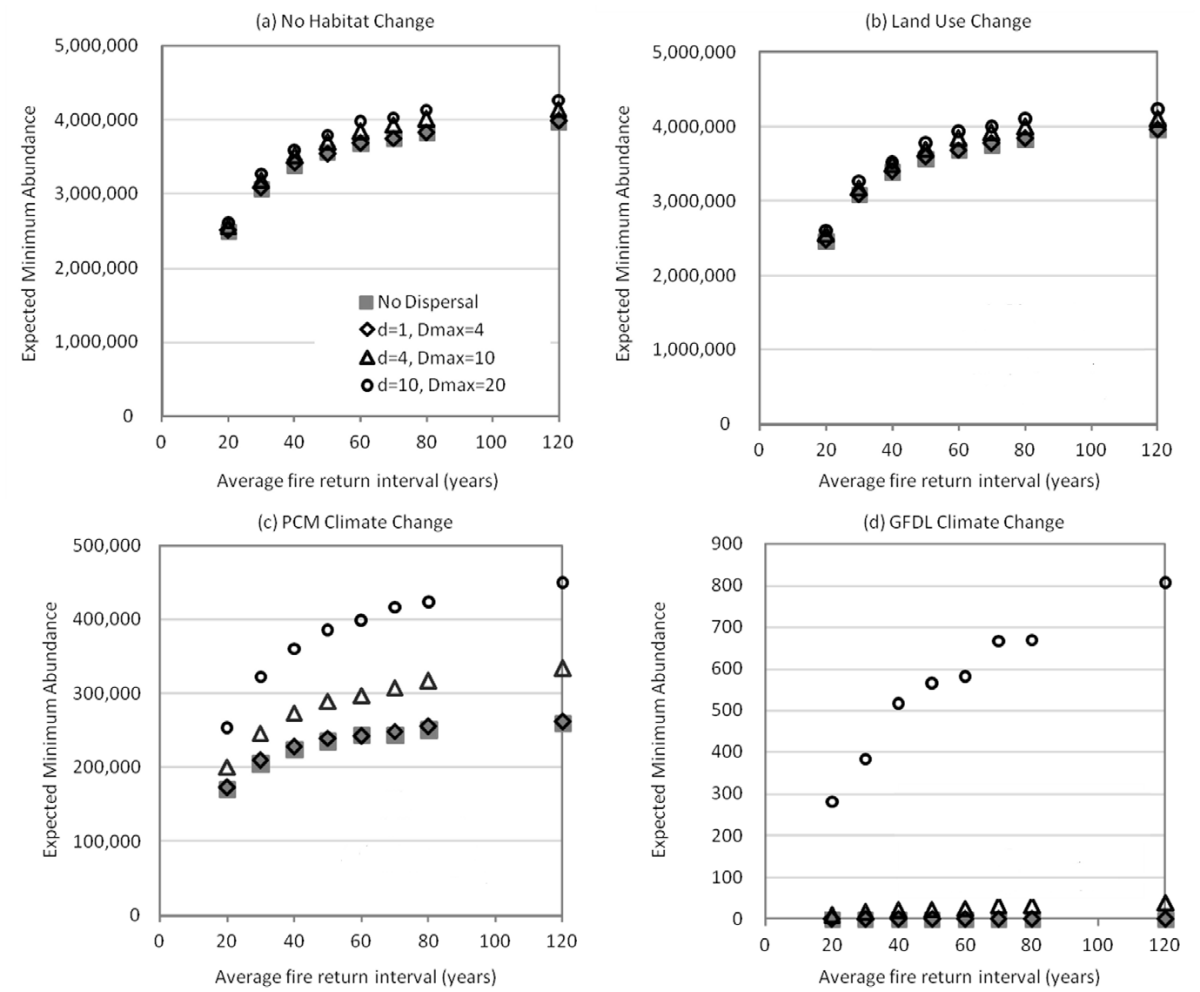
Expected minimum abundance for various dispersal, fire and habitat change scenarios. The four panels correspond to four climate and land use scenarios: (a) no habitat change, (b) land use change, (c) PCM climate change, and (d) GFDL climate change. Dispersal parameters are shown in (a) in km. The settings for the average and maximum dispersal distances *d* = 1 and *D*_max_ = 4 represent upper limits of empirically observed dispersal distances by jays. Throughout, the dispersal flow parameter is *a* = 0.01. Note the change in vertical axis units across graphs. No error bars are included because they would be smaller than the symbols on the figure. The mean of 1,000 model runs varies by less than 1% between different 1,000-run simulations.

Under all average fire return intervals, EMA was very high for the constant habitat and land use change scenarios (Fig. 3a,b), but was dramatically reduced under the PCM and GFDL scenarios (Fig. 3c,d). Steep slopes of curves in Fig. 3 indicated the importance of fire across various habitat change and dispersal scenarios. EMA under all scenarios was lower for shorter average fire return intervals. The EMA under the PCM and GFDL climate change scenarios was more sensitive to fire return interval, especially with increasing dispersal (Fig. 3c,d). When there was essentially no change in the distribution of available habitat, the EMA was relatively insensitive to dispersal parameters (Fig. 3a,b). This is not surprising given that land already occupied likely reflects *Q. engelmannii*’s dispersal abilities. In contrast, when habitat area declined and shifted under either the PCM or the GFDL scenario, the EMA was sensitive to dispersal (Fig. 3c,d). Larger average dispersal distance *d* and maximum dispersal distance *D*_max_ yielded greater EMAs, as intuition would suggest.

**Figure 4:**
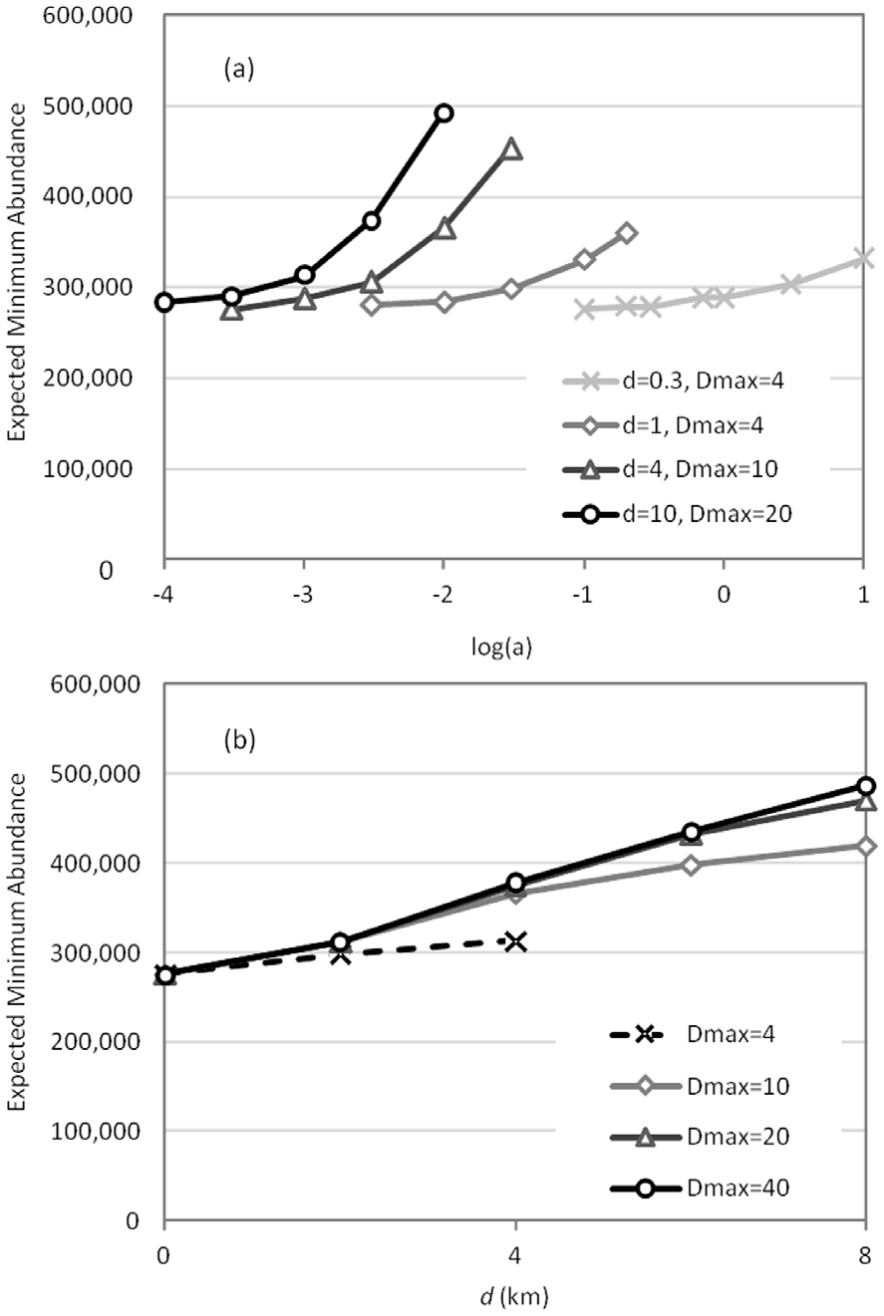
Expected minimum abundance graphed against the dispersal parameters *a* (logged) and *d* for a range of dispersal scenarios. Both panels assume the PCM scenario and the absence of fire. In (a), curves are truncated if total dispersal of acorns from a patch exceeds the number of acorns in that patch. In (b), the parameter *a* is held fixed at 0.01. The dashed line is truncated at *d* = *D*_max_.

Figure 4 shows the effects of dispersal parameters on EMA, focusing solely on the PCM climate scenario. For given *d* and *D*_max_ (average and maximum dispersal distance), and assuming no fires, the EMA increased with *a*, the distance-independent flow of acorns between patches (Fig. 4a). With *a* held fixed at 0.01, the EMA increased considerably as *d* increased from 2 to 8 km, regardless of *D*_max_ (Fig. 4b). The curves for *D*_max_ = 20 km and *D*_max_ = 40 km are nearly superimposed. (See also Appendix S3.)

**Figure 5:**
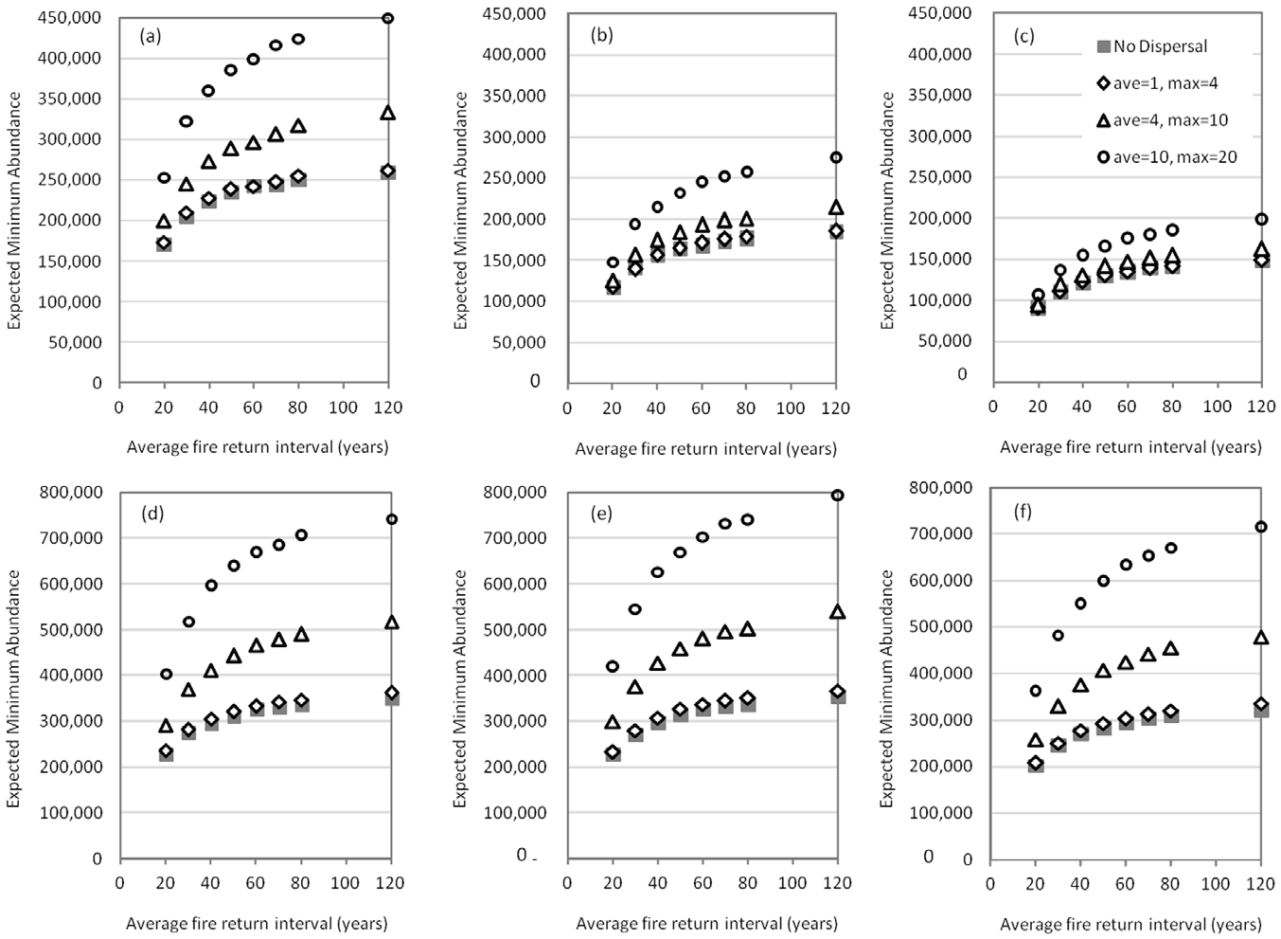
Expected minimum abundance as a function of average fire return interval for six masting-predation scenarios. Masting treatments. (a) and (d): masting probability 0.53 in a time step (or, on average, roughly every three years). (b) and (e): masting probability 0.12 in a time step (or, on average, every 16 years). (c) and (f): no masting. Predation treatments. (a), (b), and (c): ∼96% predation in masting years and ∼99% predation in non-masting years. (d), (e), and (f): ∼96% predation in all time-steps. Within each of Figures (a)–(f), dispersal distances are varied as shown on the legend in (c).

Figure 5 shows the effects of the six masting-predation scenarios on EMA. Masting was important when predation rates change with acorn production (Figs. 5a-c where the predation rate is 96% and 99% in masting and non-masting years, respectively). In these scenarios, higher masting frequency (masting probability of 0.527 in a time-step instead of 0.121) meant lower average predation rate and thus higher EMA. In contrast, when masting was not associated with lower predation (Figs. 5d-f), there was little advantage to sporadic bursts of high fecundity. The interaction between masting and predation was important.

These results were robust to sensitivity analyses (Appendix S2). Increasing each vital rate individually by 10% resulted in increased EMA of less than 10%, with the exception of adult and sapling survival (or elements (5, 5) and (4, 4) in the matrix in eqn 1) where EMA increased by 9–37% and 10–22%, respectively. Model rates were not especially sensitive to the most uncertain vital rate, or the germination parameter. Finally, the carrying capacity of the receiving patch had very little impact on the EMA under all dispersal scenarios. Regardless of the quantitative differences in EMA, the relative importance of climate change, fire frequency and dispersal were retained, suggesting that our model is robust to parameter uncertainties.

## Discussion

Maxent predicted that climatically-suitable habitat for the fire resprouter *Q. engelmannii* will decline dramatically under two future climate scenarios widely used for evaluating climate change in California. Under GFDL and PCM climate projections, assuming no migration, extinction and substantial population decline are predicted, respectively. The main difference between the two climate scenarios is the higher precipitation under PCM. The importance of water availability to *Q. engelmannii* has been demonstrated in greenhouse germination studies [31]. Field experiments show a positive correlation between *Quercus* acorn production and increased spring and summer rain [15].

Linking dynamic bioclimatic envelopes with a population model allowed us to project population size as a function of detailed, dynamic patch-to-patch dispersal. We chose the upper limit of empirically observed average and maximum dispersal distances by jays to be one and four kilometers, respectively (based on [62], [63]). There was almost no increase in EMA at these dispersal distances as compared to the no dispersal scenario. (Appendix S3 provides further details.) Altogether, our projections are pessimistic about *Q. engelmannii* persistence. Average dispersal distance and the flow of acorns were most important in mitigating population decline, suggesting that the establishment of “founder populations” depends critically on multiple propagules moving to new habitat. High acorn flow allows *Q. engelmannii* to take advantage of ephemeral habitat patches, where adequate abundances at the front of a shifting population are important for long-distance dispersal [66].

Another interpretation of our results is that a “fat-tailed” dispersal kernel would be needed to increase *Q. engelmannii* population persistence. We modeled dispersal assuming exponential decline in the fraction of acorns dispersing longer distances, based on [59]. However, that study also found that other functional forms fit the data with a high degree of statistical significance. In fact, the best fit was quadratic because there was a local peak for dispersal within a habitat patch and a long tail for dispersal between patches. Exploring additional dispersal functions and their influence on population persistence would be a fruitful future study.

Interaction between *Q. engelmannii* acorns and their predators have sizable estimated effects on population persistence. More frequent masting results in higher EMAs when relative predation rates (as a fraction of the acorn crop) are lower in masting years, but not when predation rates are the same. The first case is likely more realistic since differential acorn production in non-masting and masting years will likely keep predator populations low enough that acorn production in masting years will satiate predators’ appetites. However, there is currently minimal data on the impact of climate change on masting, thus, model studies allow us to explore potential impacts.

Predation rates are based on data collected for other *Quercus* species due to a lack of predation studies performed on *Q. engelmannii*. Use of collateral data is frequently a necessity in population modeling [53]. Although the seed predators used to parameterize our models [18], [52], [67], [68]; see Appendix S1.5) are different than those present in *Q. engelmannii* habitat, we were careful to select predators that are functionally similar to *Q. engelmannii* predators. We did not identify seedling and sapling predation rates, despite their importance [32], because they were already incorporated in the vital rates reported in [49]. These approximations were appropriate for our objective of determining how changes in masting and predation might influence population persistence as compared to other threats.

Because *Q. engelmannii*’s projected distribution of climatically-suitable habitat is centered in eastern San Diego County, which is not expected to develop substantially, land use change will likely not have a substantial direct effect on *Q. engelmannii* populations. Nonetheless, there may be a substantial indirect effect of land use change through increasing fire frequency [23], [69]. Across all scenarios, the model projects EMA to be at least 50% higher for 120-year fire return intervals than for 20-year intervals, and at least 39% higher for 60-year intervals than for 20-year intervals. Nevertheless, impacts from repeated fires are likely to be less detrimental to oak species than they are to obligate seeders [24], [70]. As a resprouter, *Q. engelmannii* can tolerate fire in many situations, though it is not suited to take advantage of fire.

Our modeling framework allows study of interactions among threats. For example, in the absence of dispersal, the EMA in landscapes with a fire return interval of 80 years is approximately 47% larger than the EMA in landscapes with a fire return interval of 20 years (under PCM climate change). With dispersal (*a*, *d*, *D*_max_)  =  (0.01, 10, 20), the corresponding increase is 67%. Masting also interacts with fire, ameliorating the negative effect of frequent fire on EMA. Assuming no masting, the EMA in landscapes with a fire return interval of 80 years is approximately 54% larger than the EMA in landscapes with fire return interval of 20 years. With masting on average every three years, the 54% increase diminishes to 47% (under PCM climate change and no dispersal). Thus, conservation strategies aimed at reducing fire would help the population more in the event of decreased masting frequency.

Although our predictions suggest that climate change will be the biggest threat to *Q. engelmannii*, we believe that our models offer considerable insights compared to SDMs alone. The preceding paragraphs discuss how abundances change 39–67% under different dispersal, fire, and masting scenarios. However, even in the absence of these different scenarios we see that projected population declines are proportionally greater than habitat losses (Fig. 2), a result we expect to be robust to different species and locations. We speculate that habitat suitability predictions underestimate the impact of climate change since they don’t account for demographic factors. Some suggest that suitability predictions be interpreted as upper bound predictions of abundance (excepting population relicts or metapopulation sinks) [71]. However, some mechanistic models which consider growth and activity rates as a function of temperature predict greater tolerance to projected climate change, and therefore less habitat decline than typical SDMs [72]. If *Q. engelmannii* tolerance, phenotypic plasticity, or adaptability to climate change is greater than we expect [73], our results may over-estimate *Q. engelmannii* population decline.

Since *Q. engelmannii* is a long-lived species with highly variable acorn production and germination, observations of these parameters over the duration of a typical empirical study (*e.g.* 10–20 years) lead to uncertainty in model parameterization. Thus, we performed sensitivity analyses on parameters in the vital rates matrix and found no change in the relative importance of climate change, dispersal, and fire frequency on population persistence (Appendix S2). Modeling long-lived species is also difficult because species may persist in a location long after the location has become unsuitable, creating an extinction debt. Because there is little data showing how survival and fecundity vary at the margins of *Q. engelmannii*’*s* distribution, we cannot assess whether this model treats such an extinction debt conservatively. If we had data on how latitudinally or elevationally varying vital rates would affect population persistence, it would be interesting to build a model where climate change acts on vital rates through time as opposed to carrying capacity. In the meantime, decreased vigor at the margins of *Q. engelmannii*’s distribution are represented by scaling the carrying capacity to SDM predicted suitability.

Our primary interest in this paper was to explore dispersal and altered species interactions (through seed predation) under climate change. To do so we created hundreds of models exploring these parameters. We did not explore sensitivity of results to additional model assumptions – additional climate models, emissions scenarios, SDMs, and SDM suitability thresholds for species presence. SDMs’ high sensitivity to model inputs is well documented in the literature [74], [75]. Given the overwhelming difference between *Q. engelmannii* abundance under PCM and GFDL scenarios, it is clear that the choice of climate model, and possibly emissions scenario, greatly impacts population projections. However, the two models used in this study represent two opposing scenarios for Southern California and are likely to bracket much of the variability in the climate models. Although Maxent has been shown to be an accurate and robust model for presence-only data [76], we speculate that the choice of Maxent has a big impact on model results given the uncertainty of SDM projection into novel environments [77], [78]. In the particular case of *Q. engelmannii*, we do not believe that small changes in the choice of SDM threshold would make a significant impact on population persistence. Continuous probability distributions for *Q. engelmannii* (Appendix S1.2) show clear boundaries between suitable and unsuitable habitat. However, if we did decrease the probability threshold, we would expect larger populations with the same ranking of population persistence under different global change scenarios.

Regardless of potential population sensitivity to different assumptions regarding climate model, emissions scenarios, SDM, and thresholds for species presence, we believe that our results are robust with regard to our research objectives. Realistic dispersal rates are not likely to mitigate population decline. However, increasing the number of propagules, and not the maximum distance of rare dispersal events, could theoretically increase abundance. Masting-dependent seed predation is predicted to have a big impact on *Q. engelmannii* populations in the event that masting becomes less frequent.

## Supporting Information

Appendix S1 **Detailed description of data and methods.**
(DOC)

Appendix S2 **Sensitivity analysis.**
(DOC)

Appendix S3 **Additional figures.**
(DOC)
