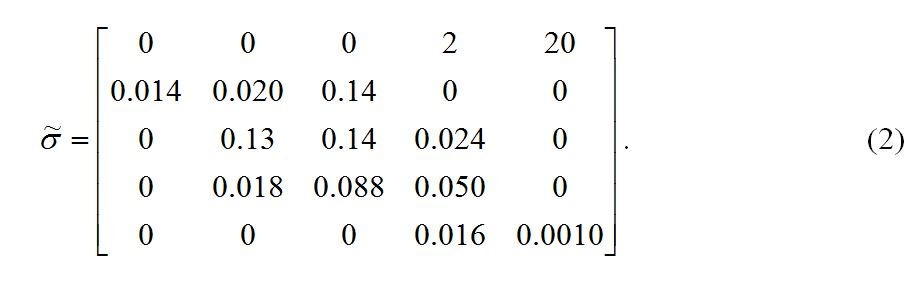# Correction: The Roles of Dispersal, Fecundity, and Predation in the Population Persistence of an Oak (*Quercus engelmannii*) under Global Change

**DOI:** 10.1371/annotation/819da56b-1c8f-445f-80fc-e8d741dc2262

**Published:** 2014-01-16

**Authors:** Erin Conlisk, Dawn Lawson, Alexandra D. Syphard, Janet Franklin, Lorraine Flint, Alan Flint, Helen M. Regan

There are errors in Equation 2. The correct version of Equation 2 can be viewed here: