# Supplementary material for: The Roles of Dispersal, Fecundity, and Predation in the Population Persistence of an Oak (Quercus engelmannii) under Global Change
Source: PLoS One. 2012 May 18;7(5):e36391. doi: 10.1371/journal.pone.0036391 (PMC3356376; doi:10.1371/journal.pone.0036391)
Supplement: Appendix S1 — Detailed description of data and methods. (DOC) [file pone.0036391.s001.doc]

**SUPPORTING INFORMATION 1. Detailed description of data and methods**

**S1.1. *Q. engelmannii***

*Q. engelmannii* is closely related to and possibly con-specific with the Mexican Blue Oak, or *Q. oblongifolia* (Nixon 2002), which resides in eastern Arizona, New Mexico, Baja California, and western Texas (Pavek 1993). *Q. englemannii* can hybridize with other California white oaks (Roberts 1995), most commonly scrub oaks (Nixon 2002). It is typically evergreen although it may lose its leaves under severe drought stress. Seeds are short-lived (no more than 15 months) and die in a fire. *Q. englemannii* seedlings provide browse for livestock and native mammals (Lathrop & Osborne 1990).

**S1.2. Habitat suitability maps**

Habitat suitability maps were discussed in the main text in the subsection **Current and future habitat distribution maps**. Table S1.1 lists the climate, soil, and terrain variables used in estimating the maps. The following text explains how these maps were transformed to metapopulation patch maps. For each (one ha) spatial cell of a map, MaxEnt predicted a continuous zero-to-one value that can be interpreted either as the probability of species presence in the cell or as the habitat suitability of the cell for the species (see Fig. S1.1). For a habitat patch in the RAMAS population model, the probability values of the constituent spatial cells were used to characterize the carrying capacity of the patch. For the population simulations, we required three types of maps: maps depicting spatial cells that were currently occupied, maps depicting currently suitable spatial cells whether or not they were occupied, and maps depicting future suitable cells at each time-step (as linearly interpolated from current and future habitat projected from the GCMs based on 2070-2099 climate). To define suitability of habitat, a minimum probability threshold (Freeman & Moisen 2008) of 0.75 was applied to the continuous predictions from MaxEnt to distinguish suitable cells (values ≥ 0.75) from unsuitable cells (values < 0.75). Adjacent suitable cells could then be aggregated into the habitat patches required for population modeling, as discussed in the main text. Suitable habitat patches <15 grid cells (15 ha) were deleted from the current and future suitable habitat maps; they were considered insufficient in size to contribute to population dynamics.

Table S1.1. Soil, terrain, and climate variables used to predict the probability of species presence.

_____________________________________________________________________________________

| **Environmental Predictor (units)** | **Source** | |
| --- | --- | --- |
| Annual precipitation (averaged over 1971-2000) | PRISM |  |
| Absolute minimum January temperature (averaged over 1971-2000) | PRISM |  |
| Absolute maximum July temperature (averaged over 1971-2000) | PRISM |  |
| Soil order | 13 categories |  |
| Soil depth (m) | STATSGO* |  |
| Soil available water capacity (cm/cm) | STATSGO* |  |
| Soil pH | STATSGO* |  |
| Slope angle (degrees) | USGS 30-m DEM |  |
| potential winter solstice solar insolation (Watt hr /m^2^) | from DEM using Solar Analyst |  |
| potential summer solstice solar insolation (Watt hr /m^2^) | from DEM using Solar Analyst |  |
| Topographic moisture index (unitless) | From DEM |  |

* STATSGO: State Soil Geographic data base for California, U.S. Department of Agriculture Natural Resources Conservation Service. [WWW document]. URL <http://gis.ca.gov/catalog/BrowseRecord.epl?id=21237>.

DEM: Digital Elevation Model; USGS: U.S. Geological Survey; Solar Analyst: an ArcView extension for modeling solar radiation at landscape scales.


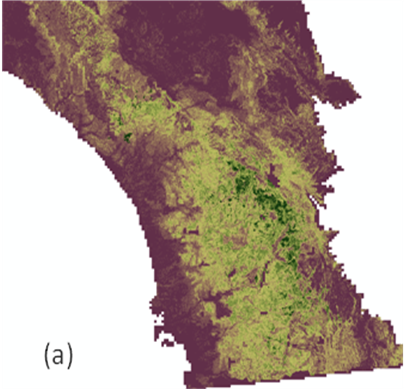

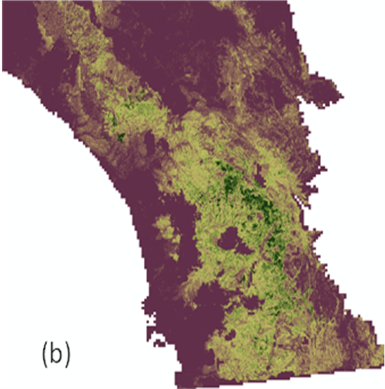

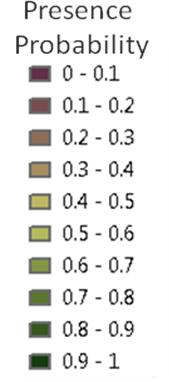


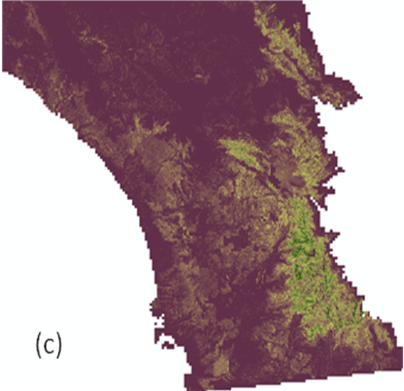

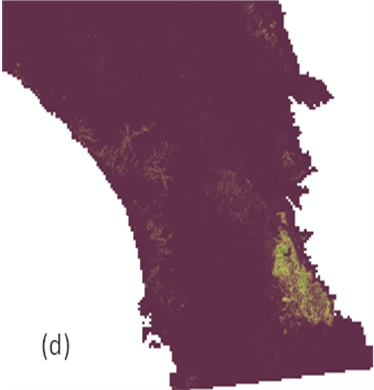


Figure S1.1. Maps of the presence probability of *Q*. *Engelmannii*. (a) Current (2000) map. (b) Future (2100) map under land use change. (c) Future map under PCM climate change. (d) Future map under GFDL climate change.

**S1.3. Demographic model**

The demographic model was described in the main text in the subsection **Demographic model structure for a single patch**. For time-steps in which there was no fire and no masting, the 5x5 matrices of means and standard deviations for the vital rates matrix were presented in eqns 1 and 2 of the main text. For convenience, they are reproduced here as Tables S1.2 and S1.3. These tables were specified from empirical findings in the literature, as discussed in detail in following sections. The mean vital rates matrix will be denoted **M** = [*μ_ij_*] in this appendix (it has no label in the main text), and the standard deviation matrix will be denoted *σ̃* (as in the main text). Neither **M** nor *σ̃* vary across patches.

For each patch and time-step, a temporary disturbance in the mean vital rates may occur in the model. There were three types of disturbances: fire, masting, and adjustment for carrying capacity violations.

*Fire:* For a time-step in which a fire occurred in a patch, the mean vital rates matrix was changed to the value in text eqn 4, reproduced as Table S1.4 here. For the next seven time-steps following the fire, the mean vital rates matrix recovered to its original value according to assumptions in Section 7 below.

*Masting:* For a time-step in which masting occurred, it occurred simultaneously for all patches. The fecundity elements *m*_14_ and *m*_15_ (for saplings and adults) from the mean vital rates matrix **M** were increased. In the main masting assumption, these two fecundities were increased by a factor of 8.2 for that one time-step. Alternate masting scenarios were also considered; see Section 5 below.

*Carrying capacity exceedance:* For a time-step in which the carrying capacity of a patch was exceeded, the mean vital rates matrix **M** was changed, for as many periods as necessary, to bring the patch back down to carrying capacity; see Section 9 for a full explanation.

Tables S1.2 to S1.4 and other parameter specifications were based on the large literature (cited below) on *Q. engelmannii*, related oak species, and various species which interact with *Q. engelmannii*. Of central importance were the mean vital rate probabilities that small seedlings, large seedlings and saplings remain in the same stage or transition to another stage, shown in gray on Tables S1.2 to S1.4. They were based on empirical studies at Camp Pendleton Marine Base (Lawson 1993). Individuals were tagged and assigned an initial stage and a final stage after two years. Three experimental treatments were imposed – no-burn, fall-burn, and spring-burn. There were three plots per treatment. Burn damage was noted for each individual. The grayed elements on Tables S1.2 and S1.4 are average rates over the three no-burn plots and the three burn-plots, respectively. The grayed elements on Table S1.3 are standard deviations over the three no-burn plots.

Table S1.2 Mean vital rates matrix **M** for a time-step with no fire and no carrying capacity exceedance. Reproduced from main text eqn 1. In a time-step when masting occurred, the two asterisked elements were increased (see Section 5 below).

|  | Acorns | Small Seedling | Large Seedling | Sapling | Tree |
| --- | --- | --- | --- | --- | --- |
| Acorns | 0 | 0 | 0 | 2* | 20* |
| Small seedling | 0.016 | 0.34 | 0.099 | 0 | 0 |
| Large seedling | 0 | 0.27 | 0.47 | 0.037 | 0 |
| Sapling | 0 | 0.0061 | 0.17 | 0.88 | 0.000001 |
| Tree | 0 | 0 | 0 | 0.025 | 0.97 |

Table S1.3 Vital rates standard deviation matrix *σ̃* for a time-step with no fire. Same as eqn 2 from the main text.

|  | Acorns | Small Seedling | Large Seedling | Sapling | Tree |
| --- | --- | --- | --- | --- | --- |
| Acorns | 0 | 0 | 0 | 2* | 20* |
| Small seedling | 0.026 | 0.020476 | 0.142544 | 0 | 0 |
| Large seedling | 0 | 0.127531 | 0.14022 | 0.023727 | 0 |
| Sapling | 0 | 0.017495 | 0.087515 | 0.05 | 0 |
| Tree | 0 | 0 | 0 | 0.015818 | 0.001 |

Table S1.4. Mean vital rates matrix for a time-step in which fire occurs. Same as eqn 4 from the main text.

|  | Acorns | Small Seedling | Large Seedling | Sapling | Tree |
| --- | --- | --- | --- | --- | --- |
| Acorns | 0 | 0 | 0 | 0 | 0 |
| Small Seedling | 0 | 0.41 | 0.13 | 0 | 0 |
| Large Seedling | 0 | 0.071 | 0.48 | 0.17 | 0 |
| Sapling | 0 | 0.0019 | 0.061 | 0.71 | 0.13 |
| Tree | 0 | 0 | 0 | 0 | 0.86 |

**S1.4. Demographic model parameterization: Tree transitions to seedlings, saplings, trees**

Consider the last column of **M**, the mean, non-fire vital rates of trees. The first element is discussed in the next section. Since trees cannot transition directly into small or large seedlings, the second and third elements were zero. In the absence of fire, adults almost never transition “backward” to saplings (Lawson 1993); hence the fourth element was set very small (0.000001). Under the RAMAS algebraic structure, this setting had to be positive to allow for a positive backward transition during a fire. In the presence of fire, adults could incur enough damage for backward transitions roughly 12.5% of the time. The fifth element of the last column, the survival probability 0.97 of an adult, was described in the main text.

**S1.5. Demographic model parameterization: Fecundity and acorn predation**

This section concerns the fourth and fifth elements of the first row of **M**, which represent the fecundity of saplings and trees in producing viable acorns. A benchmark estimate of these parameters was specified, and also variants of the estimates for use in sensitivity tests. Predation was a critical issue because a large fraction – more than 95% – of the acorns grown on trees are taken by bird, insect, and animal predators before reaching a viable state for germination.

Estimates of on-tree acorn counts were obtained from the California Acorn Survey Project (Koenig 2010, Koenig *et al*. 1994). The data consisted of 16 years (1994-2009) of 30-second visual counts of acorns for 19 *Q. englemannii* adults at the Santa Rosa Plateau in California. Fig. S1.2 shows the 16 year time series of averages and standard deviations over the 19 trees. Acorn production was highly variable from year to year, in agreement with the common generalization that most oaks mast.


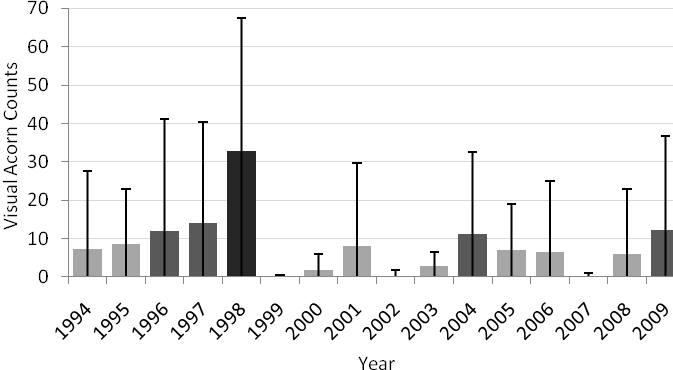


Figure S1.2. Thirty second visual acorn counts for 19 *Q. engelmannii* trees over the 16 years 1994-2009 (Koenig & Knops, personal communication). Bar heights are averages over 19 trees, and lines are corresponding standard deviations over the 19 trees. Darker gray bars are designated as masting years. The darkest bar is designated as a masting year in the alternate masting scenarios. All scenarios are described below and in Table S1.5 and S1.6.

To convert the visual on-tree counts into ground counts, the following regression from a study of central coastal California oaks (Koenig *et al.* 1994) was used:

(S1.1) ln[acorns in seed traps] = 0.17 + 0.85 ln[visual acorn counts] r^2^ = 0.82

This regression refers to the numbers of acorns that fall from trees into seed traps. Three seed traps were placed under each tree studied. Regression observations were trees, and the dependent variable refers to the sum over a tree’s three traps. A single seed trap had a surface area of 0.2 m^2^, hence a tree had a trap area of 0.6 m^2^. To convert a seed trap count per m^2^ into an overall tree count, we assumed an average canopy radius of 7.5 meters and multiplied a tree’s seed trap count by 7.5^2^ π/0.6 (dividing by 0.6 to account for the area of three seed traps). In this way, bar heights of Fig. S1.2 were converted from 30 second on-tree counts into whole-tree ground counts.

Some qualifications apply. Regarding species, Koenig *et al.* 1994 presented such regressions for *Q. agrifolia*, *Q. lobata*, and *Q. douglasii*; regressions for *Q. engelmannii* do not exist. Although *Q. agrifolia* acorns are the most similar in size to *Q. engelmannii* acorns, we used the *Quercus douglasii* regression because potential problems with the *Q. agrifolia* regression were noted by Koenig *et al.* 1994 (page 2110). For regression (S1.1), the observational unit was a tree. Koenig *et al*. 1994 observed 13 trees in each of two years, making a regression sample size of 26. In our use of the regression, our observational unit was not a single tree, but an annual average count over 19 trees. We had 16 such annual observations. It might be argued that our use of the Koenig *et al.* 1994 regression should have been further adjusted for the difference in observational unit (tree versus average of trees), but further adjustment appeared to us to be too speculative. (We could not simply do our own regression because we had no *Q. engelmanii* seed trap data, thus the conversion problem.)

The whole-tree ground counts had to be adjusted for predation. Because *Q. engelmannii* acorns are a major food source for insects, birds, and mammals, high predation rates were expected. Predation values are likely to be quite variable from year to year due to multiple factors, such as acorn timing and size (Espelta *et al*. 2009, Branco *et al*. 2002), tannin levels (Steele *et al*. 1993), habitat type (Gomez 2004, Gomez *et al*. 2003, Pulido & Diaz 2005), acorn production timing (Espelta *et al*. 2009), acorn crop size (Koenig *et al*. 2002, Espelta *et al*. 2008, Moore *et al*. 2007), habitat fragmentation (Santos & Telleria 1997), types and abundances of predators (Gomez *et al*. 2003, Borchert *et al*. 1989), and availability of alternate predator food sources, often acorns from another species of oak (Dunning *et al*. 2001). Thus, we explored multiple predation and fecundity scenarios below.

It is conventionally hypothesized that predation in masting years is a smaller fraction of the total acorn crop than in non-masting years because non-masting years will have driven down predator populations, leading large masting crops to more than satiate the predators (Koenig and Knops 2005). For a few predators, there was information on the relation of predation to acorn crop size and thus to masting. Espelta *et al*. 2008 estimated insect predation (mainly weevils, *Curculionidae*) for *Q. ilex*. Koenig *et al*. 2002 estimated insect predation for *Q. lobata*, *Q. agrifolia*, and *Q. douglasii*. Moore & Swihart 2007 estimated predation by small vertebrates for *Q. rubra* and *Q. palustris*. Koenig *et al*. 2002 estimated bird predation (mainly woodpeckers, *Melanerpes formicivorus*, and jays, *Aphelocoma coerulescens*) in mixed *Q. lobata*, *Q. agrifolia*, and *Q. douglasii* stands.

Given the nature of available studies, we divided predation into arboreal (on-tree) predation and ground predation. Arboreal predation is largely due to insects and birds (Koenig *et al*. 2002), and to a lesser degree, tree squirrels (Haas & Heske 2005). Koenig et al. 2002 commented that “predation prior to acorn fall could be up to 50% of the crop”. Eqn S1.1 already accounted for arboreal bird predation, which decreases the number of acorns that fall to the ground. Arboreal predation by insects does not decrease the number of acorns falling to the ground, instead it decreases the number of viable (capable of germination) acorns. Some falling acorns are not viable because they have been infested on the tree and will not germinate.

We used Espelta *et al.* 2008 to provide more detailed data (especially their Appendix B) on insect predation rate as a function of acorn productivity. Espelta *et al.* 2008 measured the acorn crop size and amount of insect predation on four *Q. ilex* branches from each of 15 trees during 1998-2004. We assumed that four branches accounted for 25% of a given tree's acorn production, thereby modifying four-branch acorn counts to whole-tree counts. Because acorn crop size was very small in some years, we averaged over trees to get annual average acorn crop size per tree and number of acorns predated per tree. We regressed the latter on the square root of acorn crop size, modifying the regression to recognize that the number of acorns predated cannot exceed the total acorn crop. We excluded one year of Espelta’s data for which there were fewer than five mature acorns per tree.

(S1.2) # acorns predated = min [10.4 (acorn crop size)^1/2^, acorn crop size ] r^2^ = 0.96

The curvature of this function implies that the proportion of acorns predated declines with acorn crop size, consistent with the hypothesis (two paragraphs up) that the preponderance of low crop years under masting drives down predator populations.

Next we gathered the information into two tables. The first is Table S1.5, which displays computations for six masting assumptions, or scenarios. For the next few paragraphs, we will consider only the first scenario (the top two numerical rows in Table S1.5). It is the benchmark assumption. For this benchmark, we assumed that the five largest bars on Fig. S1.2 represented masting years, and that the remaining 11 bars represent non-masting years. Thus we set the benchmark masting probability for a year at *p* = 5/16 (*p* will denote masting probability for the rest of this section). Averaging the 5 largest bar heights and the remaining 11 bar heights yielded estimates of 30 second visual acorn counts per tree per year under masting and non-masting. The averages were 1170 and 3720, as given on the “Acorns/tree arriving on the ground” column of Table S1.5.

These two acorn crop sizes, 1170 and 3720 were substituted into regression (S1.2) to estimate the numbers of acorns predated and from them the fractions of acorns arriving on the ground that were not insect infested. These fractions were 0.70 and 0.83, as given in the top two rows of Table S1.5 in the column “Fraction arriving on ground that are not insect infested” of Table S1.5. These estimates were in rough agreement with the fraction of *Q. engelmannii* acorns that had basal and apical damage in Dunning *et al.* 2001. Espelta *et al.* 2008 and Koenig *et al*. 2002 showed roughly a halving of insect predation in years with comparatively higher acorn crops. Here the fraction infested in masting years was 1–0.83 = 0.17, very roughly half of the corresponding fraction 1–0.70 = 0.30 for non-masting years.

To adjust for predation on the ground of the viable acorns arriving there, we assumed 99.25% vertebrate ground predation based on Moore & Swihart 2007 (their Figure 1, treatment 4 for north red and pin oaks). This was similar to the average of unburied and buried “oak” and “open” survival rates in unfenced plots of *Q. ilex* stands (97.7%) studied by Gomez 2004 (his Figure 1). In addition to the (100 – 99.25)% = 0.75% survival rate, we assumed that 0.41% of acorns escaped ground predation but were buried (see the estimate of buried acorns in Section 6 below), for a total of (0.75 + 0.41)% ≈ 1.2% post-dispersal ground predation survival in non-masting years. This 1.2% appears in the top row of the “Ground predation survival” column of Table S1.5.

Moore & Swihart 2007 reported that ground predation survival in masting years was 3.5 times higher than in non-masting years, based on the study of small vertebrate ground predation. We assumed that the burial rate is also 3.5 times higher, and list 3.5x1.2% = 4.2% survival in the second row of the “Ground predation survival” column of Table S1.5.

To get the final numbers of viable acorns surviving predation, for each of the first two rows, the columns “Acorns/tree arriving on ground”, “Fraction arriving on ground that are not insect infested”, and “Ground predation survival” were multiplied to get the “Surviving acorns per year” values *N* = 9.8 and *M* = 130. These final values were labeled *N* and *M* (for non-masting and masting) to prepare for notation below.

The next two rows of Table S1.5 were computed in exactly the same way except that the masting probability was set at *p* = 1/16 (instead of *p* = 5/16) and only the single tallest bar on Fig. S1.2 was designated as a masting year. The next row (the fifth row) of Table S1.5 was computed in the same way again except that the masting probability was set at *p* = 0. That is, masting was assumed not to occur at all. Thus there was only one row to consider instead of two.

This completed the top half of Table S1.5. It arrays three masting scenarios used in the text, ranging over three masting frequencies. The corresponding simulations allowed us to judge the importance of masting to the future of *Q. engelmannii*.

In the top half of Table S1.5, arboreal insect predation varied between non-masting and masting years. On the bottom half of Table S1.5, arboreal insect predation was assumed the same for non-masting acorn crop size as for masting acorn crop size. Thus, there was no difference in numbers between the top and bottom halves of the table until the columns “Fraction arriving on ground that were not insect infested” and “Ground predation survival”. In these two columns, the entries in the bottom half of the table were set the same in the first pair of rows, and the same in the second pair of rows. Thus, predation did not vary with acorn crop size between non-masting and masting years. The corresponding simulations allowed us to judge the importance of predation differences between non-masting and masting years.

Next we rearranged and extended Table S1.5 into Table S1.6. Table S1.6 has six numerical rows. They correspond to the numbered rows (1) to (6) of Table S1.5. Some of the numbers (1) to (6) represent pairs of Table S1.5 rows. The *p*, *N*, and *M* entries on Table S1.6 came from Table S1.5. The *CV* on Table S1.6 is the coefficient of variation of acorns per tree in non-masting years: the standard deviation of acorns per tree across non-masting years divided by the corresponding mean (where acorns per tree in a year was the bar height in Fig. S1.2 multiplied by 7.5^2^ π/0.6, as

Table S1.5. Fecundity rates per tree in non-masting and masting years for the six masting scenarios. In the most frequent masting scenario (probability *p* = 5/16 = 0.313 per year), the pre-predation acorn production rates were based on declaring the five tallest bars on Fig. S1.2 to be masting years. In the less frequent masting scenario (probability *p* = 1/16 = 0.0625 per year), the pre-predation acorn production rates were based on declaring the tallest bar on Fig. S1.2 to be a masting year. The predation rates reported in Table S1.6 below are products, row by row, of the two grayed values in this table.

|  | Masting probability  per year | Masting category | Acorns/tree arriving on ground | Fraction arriving on ground that were not insect infested | Ground predation survival | Surviving  acorns  per year |
| --- | --- | --- | --- | --- | --- | --- |
| Predation varies with acorn crop size | (1) *p* = 5/16 | Non-masting | 1,170 | 0.70 | 0.012 | *N* = 9.8 |
|  |  | Masting | 3,720 | 0.83 | 0.042 | *M* = 130 |
|  | (2) *p* = 1/16 | Non-masting | 1,647 | 0.74 | 0.012 | *N* = 15 |
|  |  | Masting | 6,773 | 0.87 | 0.042 | *M* = 247 |
|  | (3) *p* = 0 | Non-masting | 1,967 | 0.77 | 0.012 | *N* = 18 |
| Predation does *not* vary with acorn crop size | (4) *p* = 5/16 | Non-masting | 1,170 | 0.83 | 0.042 | *N* = 41 |
|  |  | Masting | 3,720 | 0.83 | 0.042 | *M* = 130 |
|  | (5) *p* = 1/16 | Non-masting | 1,647 | 0.87 | 0.042 | *N* = 60 |
|  |  | Masting | 6,773 | 0.87 | 0.042 | *M* = 247 |
|  | (6) *p* = 0 | Non-masting | 1,967 | 0.77 | 0.042 | *N* = 64 |

Table S1.6. shows the various parameters for *p*, *N*, *M*, *CV*, *p**, *N**, *M**, *S**, and predation rates for the six masting scenarios. Slightly different predation rates between scenarios were due to the different acorn production values input into eqn S1.2.

| Adjusted Predation Rate | | *p* | *N* | *M* | *CV* | *p** | *N** | *M** | *S** |
| --- | --- | --- | --- | --- | --- | --- | --- | --- | --- |
| Non-Masting | Masting |
| 0.992 | 0.965 | 5/16 | 9.8 | 130 | 0.71 | 0.527 | 20 | 162 | 20 |
| 0.991 | 0.963 | 1/16  0 | 15 | 247 | 0.66 | 0.121 | 30 | 271 | 28 |
| 0.991 | na | 0 | 18 | na | 0.84 | 0 | 36 | na | 43 |
| 0.965 | 0.965 | 5/16 | 41 | 130 | 0.71 | 0.527 | 82 | 187 | 82 |
| 0.963 | 0.963 | 1/16 | 60 | 247 | 0.66 | 0.121  0 | 121 | 316 | 113 |
| 0.968 | na | 0 | 64 | na | 0.84 | 0 | 127 | na | 151 |

described above). Next consider the “Adjusted Predation Rates” columns of Table S1.6, starting with the pair (0.992, 0.965) in the upper left. The 0.992, under "Non-Masting" predation, equals 1–0.70x0.012, where the 0.70 and 0.012 are the top two elements of the grayed columns of Table S1.5. The 0.965, under "Masting" predation, equals 1–0.83x0.042, where the 0.83 and 0.042 are the next two elements in the grayed columns of Table S1.5. In similar fashion, we can work down the “Adjusted predation rates” column of Table S1.6, filling in all the cells, sometimes with “na” for "not applicable".

The numbers on Table S1.5 and the columns of Table S1.6 thus far discussed were based on yearly time steps. However, the simulation model used two-year time steps. Thus, we converted critical numbers into two-year time steps. The conversion required substantial explanation and some simplifying assumptions.  The parameter *p* denotes the probability of masting in a year and the parameters *N* and *M* denote the expected final acorn production in non-masting and masting years, respectively. We assumed that the year-to-year serial correlation of acorn production was zero. The estimated serial correlation for Fig. S1.2 was *r* = 0.07, close to zero. *Q. engelmannii* may differ from oaks in Missouri which have low seed production following masting years (Sork 1993).

To determine the acorn fecundity in a two year time-step, we designated a two year time-step to be a masting time-step if there was masting in either (or both) of the two years.  Let *p**, *M**, *N**, *S** be the two year time-step parameters corresponding to the one-year parameters *p*, *M*, *N*, *S*. The conversion equations were:

*p**  =  Pr(masting at least once in two years)  =  1 – Pr(no masting in either year) =  1 – (1–*p*)^2^.

    *M**  =  E[fecundity over two years | masting in at least 1 yr]

Pr(masting in both years)2M + 2Pr(masting in one year)(*M*+*N*)

= ___________________________________________________

Pr(masting in both years) + 2Pr(masting in one year)

           =  [*p*^2^ 2*M* + *p*(1–*p*) (*M*+*N*) + (1–*p*)*p* (*M*+*N*)] / [*p*^2^ +2*p*(1–*p*)] =  2[*M*+(1–*p*)*N*]/(2–*p*).

     *N**  =  E[fecundity over two years | no masting]  =  2*N*.

*S** = 2^1/2^ x *CV* x *N**.

These conversions were used to fill in the final four columns of Table S1.6.

Now let us summarize. The first row of Table S1.6 provided benchmark parameter values for the model. The value *N** = 20 is the upper right element (tree fecundity) of the mean vital rates matrix **M** displayed in eqn 1 of the text. The element to its left (the sapling fecundity) is thus 0.1*N** = 2. (Saplings were assumed to produce 10% as many acorns as adults based on Abrahamson & Layne 2002). The value *S** = 20 appears as the upper right element of eqn 2 in the text (the standard deviation matrix) and the element to its left is 0.1*S** = 2.

The six rows of Table S1.6 can be thought of as defining a set of six fecundity scenarios by interacting three masting assumptions with two predation assumptions. The three masting assumptions were *p** = 0.527, *p** = 0.121, and *p** = 0. In the first predation assumption, predation was lower in masting time-steps (approximately 96% predation of acorns) than non-masting time-steps (approximately 99% predation). In the second predation assumption, predation was approximately 96% in both masting and non-masting time-steps. This description of the predation assumptions is rough in the sense that the 99% and 96% are only approximate and are not direct settings but rather consequences of other settings.

**S1.6. Demographic model parameterization: Germination**

The only nonzero germination was from acorn to small seedling. Its mean and standard deviation were specified as *μ*_21_ = 0.016 and *σ̃̃*_21_ = 0.026 in eqns 1 and 2 of the text. Germination was complicated by multiple factors, making germination parameters among the most uncertain in the model. Appendix B.2 presents a sensitivity analysis of *μ*_21_.

Although Snow 1972 reported on germination of *Q. engelmanii* in the greenhouse, we used field data from two studies of germination and survival to second-year seedling stage among Spanish white oak *Q. ilex*. Pulido & Diaz 2005 studied “Dehesa” and “Forested” habitats. We used their average germination rate of 0.0013. Gomez 2004 studied “open”, “oak”, “afforested”, “pine” and “shrub” habitats. We averaged germination rates for the “open” and “oak” habitat types. The study compared the germination rates of acorns buried by researchers to acorns left on the soil surface. The average germination rates were 0.074 and 0.000045 for the buried and unburied acorns. Both studies looked at germination and survival to a second-year seedling (agreeing with our use of two-year time steps). Our specification was an average of the three rates:

(S1.3) *μ*_21_ = 0.5 (0.0013) + 0.5 [ *b* (0.074) + (1–*b*) (0.000045) ] .

Here we weighted the two studies equally (the two 0.5 weights), and we weighted the buried and unburied Gomez 2004 samples *b* and 1–*b*. The weight *b* represents the fraction of potentially germinating acorns which are buried. Most of the rest of this section concerns the specification of *b*, which will depend on a collection and organization of information into Table S1.7 below. The weight *b* is important because germination rates were orders of magnitude lower for unburied than for buried acorns (Gomez 2004).

Unfortunately, there are multiple highly uncertain factors influencing the fraction of acorns that are buried and thus escape predation. Acorn burial typically occurs when jays and small mammals forget to retrieve acorns cached underground. Thus, we must specify the fractions of acorns taken, buried, and neglected by jays and small mammals. The first four columns of Table S1.7 list these specifications.

Table S1.7. Data for estimating the fraction *b* of potentially germinating acorns which were buried

|  | Fraction of Acorn Crop Taken | Fraction of Group that Buries Acorns | Fraction Cache Never Eaten | Fraction Uneaten Acorns that Remain Buried | Fraction Acorn Crop Buried |
| --- | --- | --- | --- | --- | --- |
| Birds | 0.05 | 0.25 | 0.5 | 0.5 | 0.0031 |
| Small Mammals | 0.4 | 0.5 | 0.01 | 0.5 | 0.001 |

According to exclusion experiments performed on *Quercus douglasii* in San Luis Obispo’s American Canyon, birds and mice took 5% and 40% of acorns left on the ground, respectively (Borchert et al. 1989). These fractions are recorded in the first column of Table S1.7.

Focusing on the “Birds” row of Table S1.7, we assumed that jays, the main below-ground cachers, made up one-quarter of the bird predation, as recorded in the second column of the Birds row on Table S1.7. Other bird predators, most notably woodpeckers, use arboreal caches. Jays typically neglect 50% of the acorns they cache (Borchert et al. 1989), as recorded in the third column of the Birds row on Table S1.7. Of these acorns, we assumed that half remain buried, as recorded in the fourth column of the Birds row on Table S1.7. This number is mindful of the difference in burial depth between jays (1-3 mm in sand, Borchert et al. 1989) and the experimental procedure in Gomez 2004 (buried 1-3 cm). The last entry in the Birds row on Table S1.7 is then the product of the preceding four entries in that row.

Next, focus on the Small Mammals row of Table S1.7. As already discussed, the first entry 0.4 is the fraction of acorns that fall from the tree that are taken by small mammals. We assumed that only 50% of the acorns taken are effectively buried in the sense of scatter-hoarded as opposed to larder-hoarded. Larder-hoarding, for which a few centralized and well-defended caches store the bulk of an individual’s acorns, does not facilitate germination. This 50% is recorded in the second column of the Small Mammals row. The value is highly uncertain since there are few data on the relative proportions of the two types of hoarding in *Quercus englemannii* forests (see Jenkins & Breck 1998 for discussion of hoarding in mice). Although small mammals may take a very large fraction of an acorn crop, they tend to recover acorns in underground caches at a very high rate. Squirrels in “white oak” stands (Cahalane 1942) and rodents in *Q. ilex* stands (Muñoz and Bonal 2007) have been observed to recover 99% of their cache, leaving 1%, as recorded in the third column of the Small Mammals row. Of this 1%, we assumed that 50% remain buried, as recorded in the fourth column. The last entry in the Small Mammals row is then the product of the preceding four entries.

Now turn to the weight *b*. Summing the entries in the last column of the table indicated that 0.41% of acorns taken by birds and small mammals remain buried. Moore & Swihart 2007 indicated that roughly 0.75% of acorns in unprotected areas were never taken, 30% of which were not viable due to insect predation (see above). Thus, the fraction of the potentially germinating acorn crop which was buried was *b* = 0.41/(0.41+(0.75 x 0.7)) = 0.44. Substituting *b* = 0.44 in eqn S1.3 yielded the parameter estimate *m*_21_ = 0.016. The standard deviation *σ̃̃*_21_ of the germination rate was set equal to the coefficient of variation of observed germination rates in Gomez 2004 multiplied by *m*_21_ = 0.016. The result was *σ̃̃*_21_ = 0.026.

**S1.7. Demographic model parameterization: Recovery of vital rates following a fire**

For multiple years following a fire, vital rates of *Quercus* shrubs can be affected (Keeley *et al.* 2006, Keeley & Keeley 1988). Decreased post-fire germination is likely a result of drier soil conditions, which in turn result from decreased canopy closure and decreased litter (Keeley *et al.* 2006). We assumed that these *Quercus* shrub effects would likely apply to *Q. engelmannii*, since it is a small tree sometimes occurring in shrublands. Thus we assumed that germination would be smaller after a fire. In a study of *Q. ilex*, Espelta *et al.* 1995 found that seedling germination was roughly halved for the first 15 years following coppicing. Their hypothesis was that lower canopy closure, due to selective removal of some trees, decreases germination through decreased soil moisture. We assumed a similar degree of canopy opening in burned forests where adult *Q. engelmannii* typically survive fire and resprout from the canopy crown. In the Espelta *et al.* study area, during the first 15 years following coppicing, photosynthetically active radiation decreased from roughly 40% of incoming solar radiation to 15%, and in the next five years to less than 5% (their Figure 2). They found the transition from lowered germination to full germination to be step-wise rather than smoothly gradual.

For large seedlings and saplings, canopy opening appeared to have a weak, positive effect on survival (Espelta *et al.* 1995). Directly following coppicing, the fraction of seedlings and saplings greater than five years of age was roughly 30%. In the next 15 years, the relative fraction of seedlings and saplings greater than five years of age increased to 40%. Thus, in the first 15 years after coppicing, large seedlings and saplings experience increased survival and growth compared to small seedlings. However, after this first 15 years, the fraction of seedlings and saplings dropped to less than 10%, as small seedlings were most benefitted by canopy closure.

To incorporate these changes in vital rates observed in Espelta *et al.* 1995, assuming fire has effects similar to coppicing, we decreased germination by 50% for the first 14 years (seven time steps) following a fire and increased large seedling and sapling survival and transition rates by 10%. See Table S1.8.

Table S1.8. Adjustment of the mean vital rates matrix **M** following a fire. For each of the next seven time steps following a fire, the original (pre-fire) value of **M** was adjusted. Specifically, each column of the original **M** was multiplied by the corresponding element of the first row of the following table. That is, column 1 of the original **M** was multiplied by 0.5, column 2 by 1, columns 3 and 4 by 1.1, and column 5 by 1. For the eighth and further time-steps following a fire, each column of the original **M** was multiplied by the corresponding element of the second row of the table. That is, **M** was returned to its original value. If another fire occurred within these seven time steps, the count was reset.

| Seven time steps following a fire | 0.5 | 1 | 1.1 | 1.1 | 1 |
| --- | --- | --- | --- | --- | --- |
| Eighth and further time steps following a fire | 1 | 1 | 1 | 1 | 1 |

Fecundity returned to pre-fire levels immediately following a fire time step. This assumption was based on fecundity in *Quercus geminata*, *Q. chapmannii*, *Q. myrtifolia*, and *Q. laevis* (the first two species are in section *Quercus* and the last two in section *Lobatae*), where fecundity returned to pre-fire levels within two years of a fire (Abrahamson & Layne 2002).

**S1.8. Demographic model parameterization: Population densities of initially and maximally occupied patches**

In the main text, the adult-equivalent total abundance of a patch was defined as the weighted sum of adults, saplings, large seedlings, small seedlings, and acorns using weights 1, 0.25, 0.025, 0.0025, and 0, respectively. The carrying capacity of a patch in adult-equivalent units was defined as the area of the patch in hectares times 150. The multiplier 150 was taken from studies observing adult *Q. engelmannii* at densities of 5-150 trees/ha (Lawson 1993 & Barbour 1989). The use of a carrying capacity of 150 trees per hectare translated to canopy widths of roughly 8.2 meters, which seemed a plausible width for a crowded patch. Adult-equivalency weights were based on Lawson 1993 (her Table 2), who reported average densities of 20 adults/ha, 80 saplings/ha, and 800 seedlings/ha, with a maximum of 8,200 small seedlings/ha. These densities suggested adult-equivalency weights of 20/80 = 0.25 for saplings, 20/800 = 0.025 for large seedlings, and 20/8200 ≈ 0.0025 for small seedlings. For simulation initial values, habitable patches were assigned total abundances of 90 adult-equivalents/ha, with a distribution by stage equal to an average distribution over trial runs.

**S1.9. Demographic model parameterization: Population reduction when the carrying capacity was exceeded**

At a given time-step, fecundity or dispersal might push a patch’s total abundance above its carrying capacity. Instead of forcing the abundance immediately back to capacity, we assumed a gradual, though rapid, return to capacity. For the affected patch, we reduced the survival probabilities (diagonals) and the “stage-growth” probabilities (first subdiagonals) of the baseline mean vital rates matrix **M** = [*μ_ij_*], leaving other elements unchanged. Let **M***'* = [*μ'_ij_*] denote the adjusted value. For trees, we assumed the survival probability was reduced by a fraction *c*_5_:

(a) *μ'*_55_ = *c*_5_ *μ*_55_,

For other stages, we defined:

(b) Adjusted Survival = *c_i_* (*μ_ii_* + *μ_i_*_+1_*_,i_*).

(c) Adjusted Stage-Growth = *c_i_* *μ_i_*_+1_*_,i_* / (*μ_ii_* + *μ_i_*_+1_*_,i_*).

Finally, we assumed that the adjusted values *μ'_i_*_+1_*_,i_* and *μ'_ii_* were defined by:

(d) *μ'_i_*_+1_,*_i_* = Adjusted Survival x Adjusted Stage-Growth.

(e) *μ'_ii_* = Adjusted Survival ­– *μ'_i_*_+1_,*_i_*.

Substituting (b) and (c) into (d) and (e) yielded the adjusted values:

(f) *μ'_i_*_+1_,*_i_* = *c_i_*^2^ *μ_i_*_+1_*_,i_*.

(g) *μ'_ii_* = *c_i_* [*μ_ii_* + (1–*c_i_*)*μ_i_*_+1_*_,i_*].

Using these formulas, and using (*c*_1_, *c*_2_, *c*_3_, *c*_4_, *c*_5_) = (0.1, 0.2, 0.3, 0.4, 0.5), we computed **M***'*, the adjusted vital rates matrix when carrying capacity was exceeded. It replaced **M** until the patch's total abundance no longer exceeded the carrying capacity.

**References**

Abrahamson, W.G. & Layne, J.N. (2002). Post-recovery of acorn production by four oak species in southern ridge sandhill association in south-central Florida. *Am. J. Bot.,* 89, 119-123.

Barbour, M.G. (1989).  California upland forests and woodlands. In *North American Terrestrial Vegetation*  (eds. Barbour, M.G., Billings, W.D.).  Cambridge University Press. New York, NY, p. 138.

Borchert, M.I., Davis, F.W., Michaelsen, J. & Oyler, L.D. (1989). Interactions of factors affecting seedling recruitment of Blue Oak (*Quercus douglasii*) in California. *Ecology*, 70, 389-404.

Branco, M., Branco, C., Merouani, H. & Almeida, M.H. (2002). Germination success, survival and seedling vigour of *Quercus suber* acorns in relation to insect damage. *Forest Ecol. Manage.*, 166, 159-164.

Cahalane, V.H. (1942). Caching and recovery of food by the Western Fox Squirrel. *J. Wildlife Manage.*, 6, 338-352.

Darley-Hill, S. & Johnson, W.C. (1981). Acorn dispersal by the blue jay (*Cyanocitta cristata*). *Oecologia*, 50, 231-232.

Dunning, C.E., Paine, T.D. & Redak, R.A. (2001). Insect-oak interactions with Coast Live Oak (*Quercus agrifolia*) and Engelmann Oak (*Q. engelmannii*) at the acorn and seedling stage. USDA Forest Service General Technical Report PSW-GTR-184.

Espelta, J.M., Bonal, R. & Sanchez-Humanes, B. (2009). Pre-dispersal acorn predation in mixed oak forests: interspecific differences are driven by the interplay among seed phenology, seed size and predator size. *J. Ecol.*, 97, 1416-1423.

Espelta, J.M., Cortez, P., Molowny-Horas, R., Sanchez-Humanes, B. & Retana, J. (2008). Masting mediated by summer drought reduces acorn predation in Mediterranean oak forests. *Ecology*, 89, 805-817.

Espelta, J.M., Riba, M. & Retana, J. (1995). Patterns of seedling recruitment in West-Mediterranean *Quercus ilex* forests influenced by canopy development. *J. Veg. Sci.*, 6, 465-472.

Freeman, E.A. & Moisen, G.G. (2008).  A comparison of the performance of threshold criteria for binary classiﬁcation in terms of predicted prevalence and kappa. *Ecol. Model.*, 217, 48-58.

Gomez, J.M. (2004). Importance of microhabitat and acorn burial on *Quercus ilex* early recruitment: non-additive effects on multiple demographic processes. *Plant Ecol.*, 172, 287-297.

Gomez, J.M., Garcia, D. & Zamora, R. (2003). Impact of vertebrate acorn and seedling-predators on a Mediterranean *Quercus pyrenaica* forest. *Forest Ecol. Manag.*, 180, 125-134.

Haas, J.P. & E.J. Heske. (2005). Experimental study of the effects of mammalian acorn predators on red oak survival and germination. *J. Mammal.*, 86, 1015-1021.

Jenkins, S.H. & Breck, S.W. (1998). Differences in food hoarding among six species of Heteromyid rodents. *J. Mammal.*, 79, 1221-1233.

Keeley, J.E., Fotheringham, C.J. & Baer-Keeley, M. (2006). Demographic patterns of post-fire regeneration in Mediterranean-climate shrublands of California. *Ecol. Monogr.*, 68, 524-530.

Keeley, J.E. & Keeley, S.C. (1988). Chaparral. In: *North American Terrestrial Vegetation* (eds. M.G. Barbour and W.D. Billings). Cambridge University Press. New York, NY, 165-207.

Koenig, W.D. (2010). Acorn Survey Project. [WWW document]. URL <http://www.nbb.cornell.edu/wkoenig/wicker/CalAcornSurvey.html>

Koenig, W.D. & Knops, J.M.H. (2005). The mystery of masting in trees. *Am. Sci.*, 93, 340-347.

Koenig, W.D., Knops, J.M.H. & Carmen, W.J. (2002). Arboreal seed removal and insect damage in three California oaks. USDA Forest Service General Technical Report PSW-GTR-184.

Koenig, W.D., Knops, J.M.H., Carmen, W.J., Stanback, M.T. & Mumme, R.L. (1994). Estimating acorn crops using visual surveys. *Can. J. For. Res.*, 24, 2105-2112.

Lawson, D. (1993). Effects of Fire on Stand Structure of Mixed *Quercus agrifolia* and *Quercus engelmannii* Woodlands. Master’s Thesis. San Diego State University.

Moore, J.E., McEuen, A.B., Swihart, R.K., Contreras, T.A. & Steele, M.A. (2007). Determinants of seed removal distance by scatter-hoarding rodents in deciduous forests. *Ecology*, 88, 2529-2540.

Moore, J.E. & Swihart, R.K. (2007). Importance of fragmentation-tolerant species as seed dispersers in disturbed landscapes. *Oecologia*, 151, 663-674.

Muñoz, A. & Bonal, R. (2007). Rodents change acorn dispersal behavior in response to ungulate presence. *Oikos*, 116, 1631-1638.

Nixon, K.C. (2002). The oak (*Quercus*) biodiversity of California and adjacent regions. USDA Forest Service General Technical Report PSW-GTR-184.

Pavek, D.S. (1993). Quercus oblongifolia. In: Fire Effects Information System (Online). U.S. Department of Agriculture, Forest Service, Rocky Mountain Research Station, Fire Sciences Laboratory (Producer). [WWW document] URL http://www.fs.fed.us/database/feis/

Pulido, F.J. & Diaz, M. 2005. Regeneration of a Mediterranean oak: a whole-cycle approach. *Bioscience*, 12, 92-102.

Roberts, F.M. (1995). *Illustrated Guide to the Oaks of the Southern California Floristic Province*. F.M. Roberts Publications, Encinitas, CA.

Santos, T. & Telleria, J.L. (1997). Vertebrate predation on Holm Oak, *Quercus ilex*, acorns in a fragmented habitat: effects on seedling recruitment. *Forest Ecol. Manag.*, 98, 181-187.

Snow, G.E. (1972). Some factors controlling the establishment and distribution of *Quercus agrifolia* and *Quercus engelmannii* Greene in certain Southern California oak woodlands. PhD Thesis in Botany. Oregon State University.

Sork, V.L. (1993). Evolutionary ecology of mast-seeding in temperate and tropical oaks (*Quercus* spp.). *Vegetatio*, 107/108, 133-147.

Steele, M.A., Knowles, T., Bridle, K., & Simms, E.L. (1993). Tannins and partial consumption of acorns: implications for dispersal of oaks by seed predators. *Am. Midl. Nat.*, 130, 229-238.
