# Supplementary material for: The Roles of Dispersal, Fecundity, and Predation in the Population Persistence of an Oak (Quercus engelmannii) under Global Change
Source: PLoS One. 2012 May 18;7(5):e36391. doi: 10.1371/journal.pone.0036391 (PMC3356376; doi:10.1371/journal.pone.0036391)
Supplement: Appendix S2 — Sensitivity analysis. (DOC) [file pone.0036391.s002.doc]

**SUPPORTING INFORMATION 2. Sensitivity analysis**

**S2.1. Sensitivity of EMA to vital rates**

The sensitivities of the EMA to all-else-equal 10% increases in vital rates are considered on Table S2.1. Each increase was considered for two fire return intervals (fires every 20 years and no fires) and three dispersal scenarios (no dispersal, *d* = 1 and *D*_max_ = 4, and *d* = 10 and *D*_max_ = 20) for the PCM climate change scenario. See the Table S2.1 caption for more details.

Table S2.1. Sensitivity of EMA to 10% increases in mean vital rates

|  | 20 year fire return interval | | | No fires | | |
| --- | --- | --- | --- | --- | --- | --- |
|  | No dispersal | *d*=1, *D*_max_=4 | *d*=10, *D*_max_=20 | No dispersal | *d*=1, *D*_max_=4 | *d*=10, *D*_max_=20 |
| *μ*21: germination | 1.11 | 1.09 | 1.09 | 1.07 | 1.07 | 1.10 |
| *μ*22: small seedling survival | 1.07 | 1.05 | 1.06 | 1.04 | 1.04 | 1.07 |
| *μ*32: small to large seedling | 1.08 | 1.06 | 1.09 | 1.05 | 1.04 | 1.09 |
| *μ*42: small seedling to sapling | 1.02 | 1.01 | 1.00 | 1.00 | 1.00 | 1.01 |
| *μ*23: large to small seedling | 1.02 | 1.01 | 1.00 | 1.01 | 1.00 | 1.02 |
| *μ*33: large seedling survival | 1.08 | 1.07 | 1.09 | 1.05 | 1.04 | 1.08 |
| *μ*43: large seedling to sapling | 1.06 | 1.05 | 1.06 | 1.03 | 1.04 | 1.05 |
| *μ*34: sapling to large seedling | 1.00 | 1.00 | 0.99 | 1.01 | 1.02 | 1.01 |
| *μ*44: sapling survival* | 1.13 | 1.13 | 1.15 | 1.11 | 1.10 | 1.22 |
| *μ*54: sapling to adult | 1.04 | 1.02 | 1.03 | 1.02 | 1.00 | 1.04 |
| *μ*55: adult survival* | 1.27 | 1.26 | 1.32 | 1.02 | 1.01 | 1.09 |
| *μ*14: sapling fecundity | 1.03 | 1.01 | 1.01 | 1.02 | 1.01 | 1.02 |
| *μ*15: adult fecundity | 1.08 | 1.05 | 1.09 | 1.06 | 1.05 | 1.08 |

Each tabled entry is a sensitivity of the EMA to an all-else-equal 10% increase in an underlying vital rate parameter (relative to the mean parameter values of text eqn 1), measured at a particular fire return interval and for a particular dispersal scenario, as shown on the table heading. Each tabled value is the ratio of the EMA with the 10% increase to the EMA without the increase. All ratios assume PCM climate change. A large tabled entry indicates an important parameter to the model.

**S2.2. Germination**

Empirical studies suggest that *Quercus* germination is highly variable. Multiple factors influence germination rates, including: climate (Snow 1991, 1972), microhabitat (Gomez 2004, Pullido & Diaz 2005), seed size (Darley-Hill & Johnson 1981), and time since last disturbance (Keeley *et al.* 2006, Keeley & Keeley 1988, Espelta *et al.* 1995, Valbuena & Tarrega 1998). For example, above 100 bars of soil moisture tension, 50% of newly emerged *Q. engelmannii* seedlings will die (Snow 1972).

In the absence of data adequate to study germination directly, we ran simulations to determine the sensitivity of model results to the key germination parameter, which is the vital rate *μ*_21_ in eqn 1 of the main text. Would changes to *μ*_21_ substantially change the expected mean abundances of *Q. engelmannii* under alternative scenarios? Each scenario considered was a combination of a dispersal setting, a climate setting, and a fire setting from the following possibilities.

Dispersal: (i) no dispersal. (ii) *a* = 0.01, *d* = 1, *D*_max_ = 4. (iii) *a* = 0.01, *d* = 10, *D*_max_ = 20.

Climate: (i) no change. (ii) PCM change.

Fire: 20 to 120 year average fire return interval.

For each dispersal-climate-fire scenario, we considered germination rates that are 0.5, 1.0, and 1.5 times the setting *μ*_21_ = 0.016 from eqn 1 of the main text. Fig. S2.1 displays results. Increased germination rates increased expected minimum abundance, but do not alter the relative importance of dispersal, climate, and fire frequency.


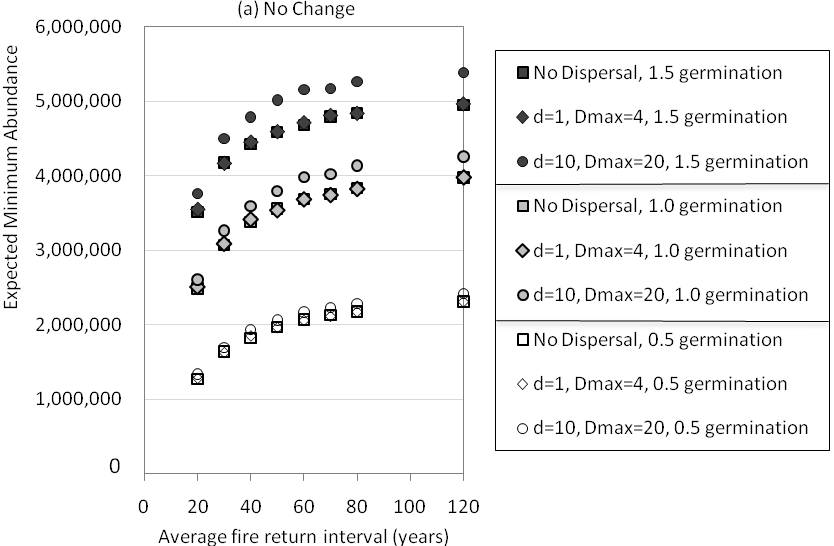

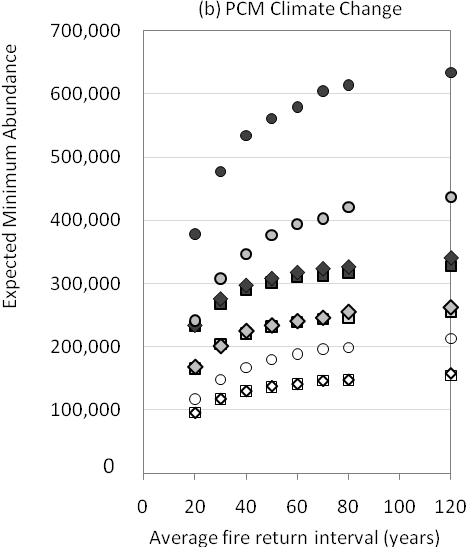


Figure S2.1. Expected minimum abundance for germination rates of 0.5 (open symbols), 1.0 (light gray symbols), and 1.5 (dark gray symbols) times the germination rate used in the main text. Results are shown for a variety of fire return intervals, and for (a) no climate change and (b) PCM climate change. Note the change in scale on the y-axis between (a) and (b).

**S2.3. Dispersal**

As in the main text, let *M_ij_* denote the fraction of acorns in a time period which disperse from a source patch *i* to a recipient patch *j*, and let *D_ij_* be the distance from patch *i* to patch *j*. The primary dispersal assumption, subject to a condition that the recipient patch is not too far from the source patch, has been that *M_ij_* depends negatively on *D_ij_* according to the equation *M_ij_* = *a* exp(–*D_ij_* /*d*), where *a* and *d* are positive parameters. Thus, dispersal of a particular acorn to a nearby patch is more likely than dispersal to a distant patch. We here consider the added hypothesis, subject to a condition that the recipient patch is not too large, that *M_ij_* depends positively on the carrying capacity *K_j_* of the recipient patch. The equation is expanded to *M_ij_* = (*a*/*P*) *K_j_* exp(–*D_ij_* /*d*), where *P* is an additional positive parameter. Under this equation, dispersal of a particular acorn is more likely to a larger patch than to an equally distant smaller patch.

Now add the further condition that the recipient patch is not too large. The final *M_ij_* equation is:

*M_ij_* = *a* min(1, *K_j_* /*P*) exp(–*D_ij_* /*d*) if *D_ij_* < *D*_max_,

(S2.1)

*M_ij_* = 0 if *D_ij_* ≥ *D*_max_.

The second line states that no dispersal occurs beyond a maximal distance *D*_max_. The first line states the condition, embodied in the expression min(1, *K_j_* /*P*), that the dependence of *M_ij_* on *K_j_* “turns off” when *K_j_* > *P*. Thus, the new parameter *P* reflects the strength of the carrying capacity mechanism. When *P* = 0, the mechanism disappears, and eqn S2.1 reverts to the form used in the main text. When *K_j_* > *P* *>* 0, eqn S2.1 becomes *M_ij_* = (*a*/*P*) *K_j_* exp(–*D_ij_* /*d*), which states that the fraction of acorns dispersing from patch *i* to patch *j* is decreasing in the distance *D_ij_* and increasing in the capacity *K_j_*. The effects are multiplicative and thus interact. We considered this particular functional form because the RAMAS platform was programmed to accommodate it, and it seemed plausible, although there are no studies we know of discussing patch-size dependent dispersal. In the main text, *P* = 1 was assumed. Here we report tests of the sensitivity of model results to different values of *P*.

For the tests, we considered the two values *P* = 2000 and *P* = 4000, in addition to the *P* = 1 setting of the main text. Will changes in *P* substantially change expected mean abundances under alternative scenarios? Each scenario was a combination of a setting for the dispersal parameters (*a*, *d*, *D*_max_), a climate setting, and a fire setting:

Dispersal: (i) *a* = 0.01, *d* = 4, *D*_max_ = 10. (ii) *a* = 0.01, *d* = 10, *D*_max_ = 20.

Climate: (i) no change, (ii) PCM.

Fire: 20 to 120 year average fire return interval.

The results are shown in Fig. S2.2. As with the germination sensitivity analysis, there was no change in the relative importance of dispersal, climate, or fire frequency. However, the expected minimum abundance decreases with increasing *P*.


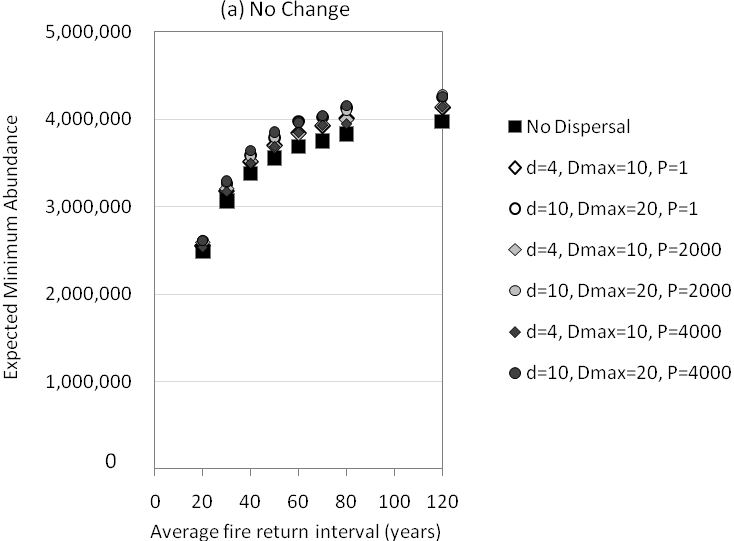

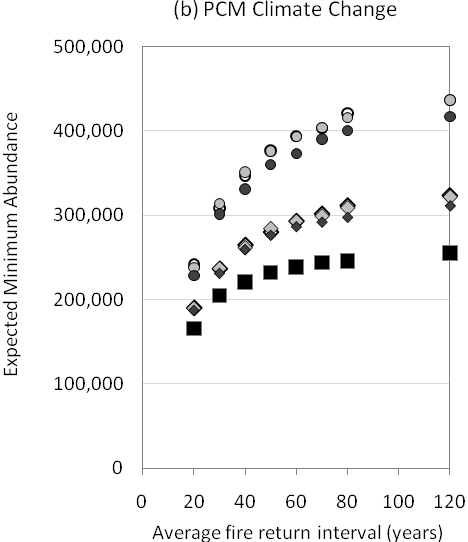


Figure S2.2. Expected minimum abundance for alternative dispersal scenarios (see the legend), for a variety of fire return intervals, and for (a) no climate change and (b) PCM climate change. Note the change in scale on the y-axis between (a) and (b). Although the expected minimum abundance decreases as *P* increases, there is no change in the relative importance of dispersal, climate, and fire frequency.

**References**

Darley-Hill, S. & Johnson, W.C. (1981). Acorn dispersal by the blue jay (*Cyanocitta cristata*). *Oecologia*, 50, 231-232.

Espelta, J.M., Riba, M. & Retana, J. (1995). Patterns of seedling recruitment in West-Mediterranean *Quercus ilex* forests influenced by canopy development. *J. Veg. Sci.*, 6, 465-472.

Gomez, J.M. (2004). Importance of microhabitat and acorn burial on *Quercus ilex* early recruitment: non-additive effects on multiple demographic processes. *Plant Ecol.*, 172, 287-297.

Keeley, J.E., Fotheringham, C.J. & Baer-Keeley, M. (2006). Demographic patterns of post-fire regeneration in Mediterranean-climate shrublands of California. *Ecol. Monogr.*, 68, 524-530.

Keeley, J.E. & Keeley, S.C. (1988). Chaparral. In: *North American Terrestrial Vegetation* (eds. M.G. Barbour and W.D. Billings). Cambridge University Press. New York, NY, 165-207.

Pulido, F.J. & Diaz, M. 2005. Regeneration of a Mediterranean oak: a whole-cycle approach. *Bioscience*, 12, 92-102.

Snow, G.E. (1991). Germination Characteristics of Engelmann Oak and Coast Live Oak from the Santa Rosa Plateau, Riverside County, California. USDA Forest Service General Technical Report PSW-126.

Snow, G.E. (1972). Some factors controlling the establishment and distribution of *Quercus agrifolia* and *Quercus engelmannii* Greene in certain Southern California oak woodlands. PhD Thesis in Botany. Oregon State University.

Valbuena, L. & Tarrega, R. (1998). The influence of heat and mechanical scarification on the germination capacity of *Quercus pyrenaica* seeds. *New Forest.*, 16, 177-183.
