# Supplementary material for: The Roles of Dispersal, Fecundity, and Predation in the Population Persistence of an Oak (Quercus engelmannii) under Global Change
Source: PLoS One. 2012 May 18;7(5):e36391. doi: 10.1371/journal.pone.0036391 (PMC3356376; doi:10.1371/journal.pone.0036391)
Supplement: Appendix S3 — Additional figures. (DOC) [file pone.0036391.s003.doc]

**SUPPORTING INFORMATION 3. Additional figures**

The following figures show the importance of different land use and climate scenarios (Fig. S3.1) and the importance of the dispersal parameters *a* and *d* across different fire return intervals (Fig. S3.2-S3.4).


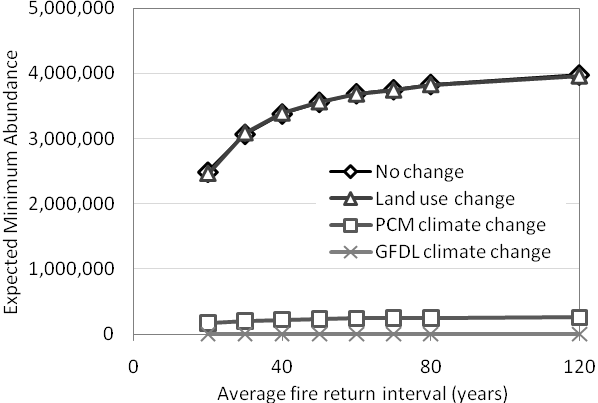


Figure S3.1. Expected minimum abundance as a function of the average fire return interval for each of four scenarios: GFDL climate scenario, PCM climate scenario, land use change, and no change, where suitable habitat remains unchanged until 2100. These scenarios do not include dispersal.


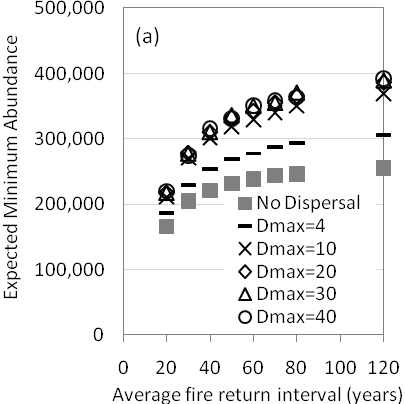

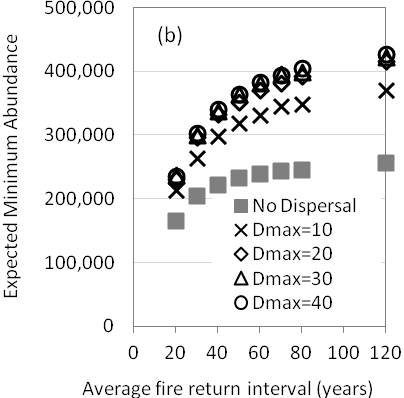


Figure S3.2. Expected minimum abundance as a function of average fire return interval for different *D*_max_ scenarios: (a) *d* = 4 km and *a* = 0.02, and (b) *d* = 8 km and *a* = 0.01.


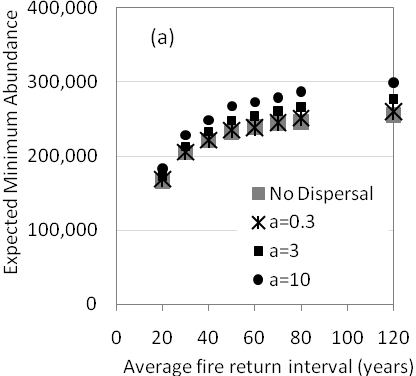

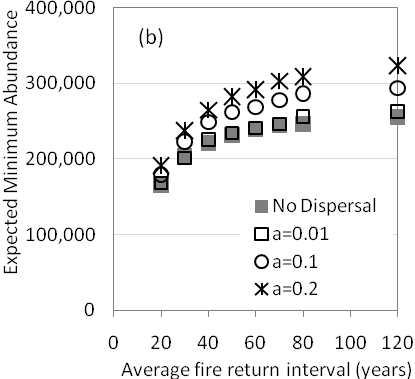


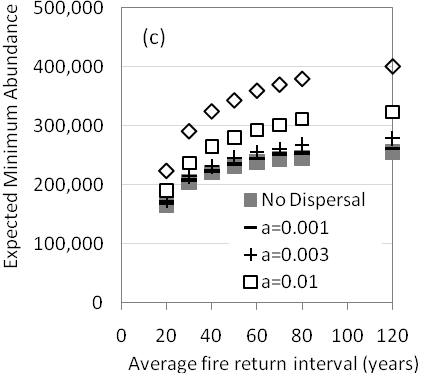

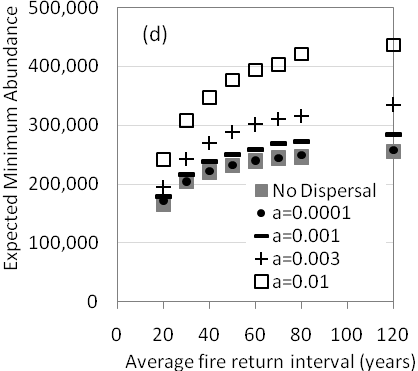


Figure S3.3. Expected minimum abundance under the PCM scenario as a function of average fire return interval for different values of *a*. The legend shows *a* values, where the parameter values are as follows: (a) *d* = 0.3 km and *D*_max_ = 4 km, (b) *d* = 1 km and *D*_max_ = 4 km, (c) *d* = 4 km and *D*_max_ = 10 km, and (d) *d* = 10 km and *D*_max_ = 20 km. As dispersal distances increase from (a) to (d), lower values of *a* result in increased expected minimum abundance.


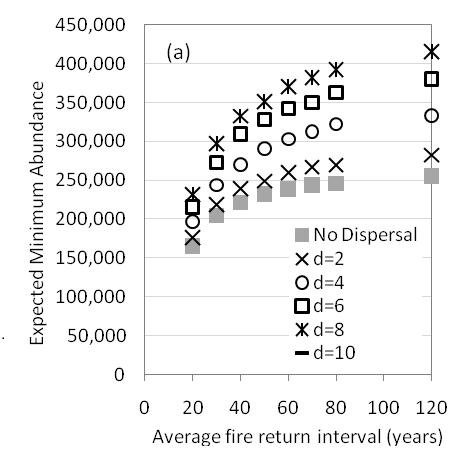

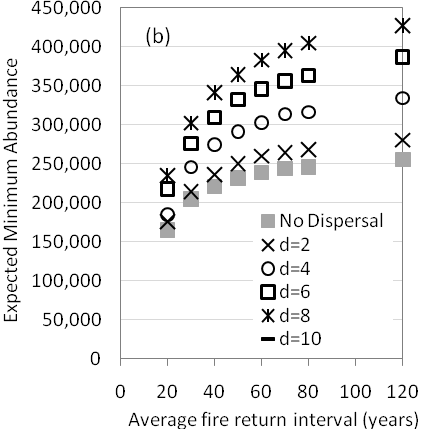


Figure S3.4. Expected minimum abundance as a function of average fire return interval for different average dispersal scenarios: (a) *D*_max_ = 20 km, and (b) *D*_max_ = 40 km. For both figures, *a* = 0.01.
